# Poly(amino acid) based fibrous membranes with tuneable *in vivo* biodegradation

**DOI:** 10.1371/journal.pone.0254843

**Published:** 2021-08-13

**Authors:** Kristof Molnar, Constantinos Voniatis, Daniella Feher, Gyorgyi Szabo, Rita Varga, Lilla Reiniger, David Juriga, Zoltan Kiss, Eniko Krisch, Gyorgy Weber, Andrea Ferencz, Gabor Varga, Miklos Zrinyi, Krisztina S. Nagy, Angela Jedlovszky-Hajdu

**Affiliations:** 1 Laboratory of Nanochemistry, Department of Biophysics and Radiation Biology, Semmelweis University, Budapest, Hungary; 2 Department of Food, Agricultural and Biological Engineering, College of Food, Agricultural, and Environmental Sciences, The Ohio State University, Wooster, OH, United States of America; 3 Department of Surgical Research and Techniques, Semmelweis University, Budapest, Hungary; 4 1^st^ Department of Pathology and Experimental Cancer Research, Semmelweis University, Budapest, Hungary; 5 Department of Polymer Engineering, Faculty of Mechanical Engineering, Budapest University of Technology and Economics, Budapest, Hungary; 6 Biomechanical Research Center, Faculty of Mechanical Engineering, Budapest University of Technology and Economics, Budapest, Hungary; 7 Department of Oral Biology, Semmelweis University, Budapest, Hungary; Shanghai Jiao Tong University Medical School Affiliated Ruijin Hospital, CHINA

## Abstract

In this work two types of biodegradable polysuccinimide-based, electrospun fibrous membranes are presented. One contains disulfide bonds exhibiting a shorter (3 days) *in vivo* biodegradation time, while the other one has alkyl crosslinks and a longer biodegradation time (more than 7 days). According to the mechanical measurements, the tensile strength of the membranes is comparable to those of soft the connective tissues and visceral tissues. Furthermore, the suture retention test suggests, that the membranes would withstand surgical handling and *in vivo* fixation. The *in vivo* biocompatibility study demonstrates how membranes undergo *in vivo* hydrolysis and by the 3^rd^ day they become poly(aspartic acid) fibrous membranes, which can be then enzymatically degraded. After one week, the disulfide crosslinked membranes almost completely degrade, while the alkyl-chain crosslinked ones mildly lose their integrity as the surrounding tissue invades them. Histopathology revealed mild acute inflammation, which diminished to a minimal level after seven days.

## 1. Introduction

Polymer hydrogels are three-dimensional polymer networks that contain a large amount of aqueous solution. Hydrogels possess the properties of both solid and liquid materials: they can maintain their shape as solids do, yet small molecules for example drugs, can migrate through them by diffusion (as they would do in fluids). Furthermore, due to their high water content, they resemble the mammalian soft tissues, therefore, hydrogels are commonly used in clinical practice and experimental medicine in a wide range of applications including diagnostics, drug delivery, regenerative medicine [1–5]. However, hydrogels are typically fragile, brittle resulting in breaking upon bending, suturing or other basic handling maneuvers during surgeries, severely limiting their applicability as implants [6, 7].

Fibrous hydrogel membranes are soft materials composed of sub-micron diameter hydrogel fibers bundled together that create a loose and flexible sheet. While still being considered as hydrogels, fibrous hydrogel membranes have additional advantages. Not only these membranes possess the favorable features of hydrogels, but their structure is similar to the extracellular matrix found ubiquitously around almost every human cell. Therefore, these membranes are exceptional candidates for cell cultivation or tissue regeneration applications. Furthermore, being composed of fibers, the material should be more flexible, but also more resistant to the damage caused by the sutures due to the free movement and bending of their fibers.

Fibrous membranes have been extensively researched for a wide variety of fields [8] including biomaterials [9, 10]. One method for polymer fiber preparation is electrospinning, where polymer fibers are created under the effect of a strong electrical field [11]. By introducing a crosslinking reaction to electrospinning, the aforementioned gel fibers can be obtained, which, without chemical or enzymatic degradation, will not dissolve after being immersed in a solution but only absorb the surrounding fluid [12]. It is important to note here that in gel fibers crosslinks are between polymer chains inside the fibers and not between fibers. Typically, one of the two following implementation methods is utilized for the synthesis of crosslinked fibrous membranes (gel fibers). In post-electrospinning methods, the fibrous membrane (having the crosslinking agent already in the polymer solution) is electrospun first and the crosslinks are formed subsequently in a chemical reaction [13–15]. By utilizing this method, however, the number of crosslinks cannot be properly regulated. On the other hand, in reactive electrospinning, the chemical crosslinking reaction takes place during the fiber formation. Typically, a UV active reagent is added to the polymer that under the effect of UV light will auto-crosslink the polymer chains inside the fibers. Although by utilizing this method the number of crosslinks can be regulated, most of these UV active reagents are toxic [16, 17].

Poly(aspartic acid) (PASP) is a synthetic biocompatible polymer composed of aspartic acid molecules interconnected by peptide bonds and thus exhibits a peptide-like chemical structure, which ensures its biodegradability [18–24]. PASP based hydrogels are promising materials for tissue engineering and cell cultivation. Juriga *et al*. displayed how MG-63 cells could not just attach and proliferate on PASP based hydrogels, but were also able to grow inside the gels and establish 3D colonies [25]. Nevertheless, PASP based hydrogels are simply too fragile for conventional implantation. In addition, although the fabrication these membranes was proven feasible, [26, 27] as promising as these materials are, no information is available regarding any implantation attempts, *in vivo* biocompatibility or a biodegradability profile.

**Fig 1:**
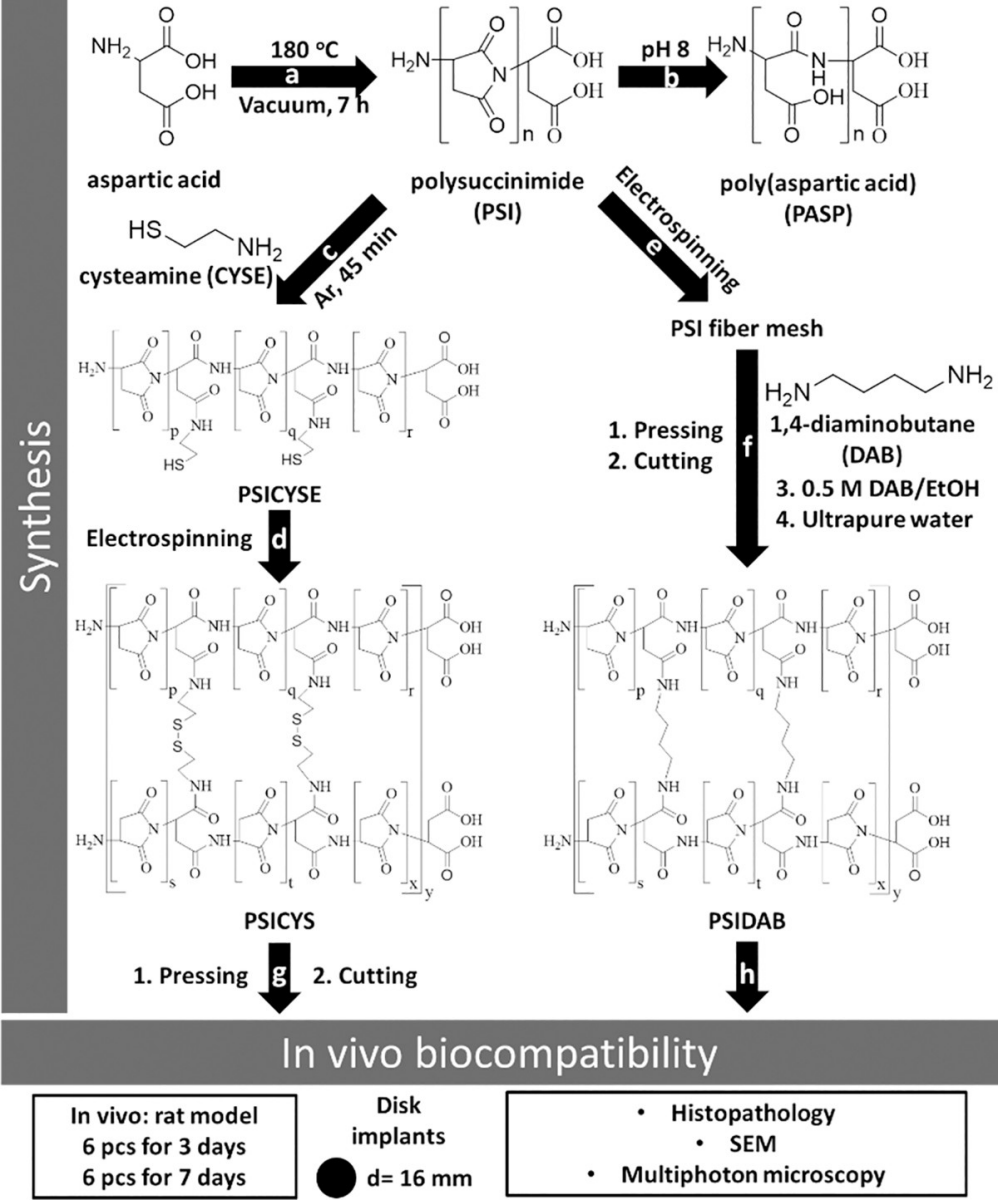
Schematic of the synthesis and investigation of materials subjected in this paper: synthesis of PSI (a); hydrolysis of PSI (b); modification of PSI with cysteamine (c); reactive electrospinning of cysteamine modified PSI (d); electrospinning of PSI (e); pressing, cutting and heterogenous crosslinking of polysuccinimide fibers with 1,4-diaminobuthane (f); pressing and cutting of PSICYS samples and implantation in a rat animal model (g); implantation of PSIDAB disks in a rat animal model (h).

PASP can be prepared from its anhydride, polysuccinimide (PSI) via hydrolysis under mild alkaline conditions (Fig 1) [28]. Unlike PASP, PSI is a reactive polymer, therefore, it can be easily modified at room temperature by nucleophilic reagents such as primary amines, giving PSI a major advantage compared to other synthetic biocompatible and biodegradable polymers. This enables us to synthesize a large variety of PSI derivatives with adjustable properties for different applications (Fig 1), which can be later on hydrolyzed to the corresponding PASP derivatives either *in vitro* or *in vivo* [19–24, 29–31]. By utilizing multifunctional amines for crosslinking, advanced functional PSI gels or with their hydrolysis PASP hydrogels can be created [28].

In this work we present the fabrication and characterization two PSI based crosslinked fibrous membranes (referred to as fibrous membranes from now on) which can be implanted by conventional surgical techniques while also retaining their hydrolytic and enzymatic degradability. The two types of membranes were fabricated having different chemical compositions: a disulfide crosslinked one intended for fast biodegradation and an alkyl-chain crosslinked one with possibly longer biodegradation time, both intended for tissue engineering. These samples were implanted under the skin in PSI form to see if they can hydrolyze and form PASP based samples, how fast the hydrolysis occurs, how the surrounding tissue reacts to these changes and to see the short-term biodegradability of the membranes. The biocompatibility/biodegradability was comprehensively investigated.

## 2. Experimental

### 2.1. Materials

L-aspartic acid (Sigma-Aldrich, UK), cysteamine (CYSE) (Sigma-Aldrich, UK), dimethylformamide (DMF) (VWR International, USA), dimethylsulfoxide (DMSO) (Sigma-Aldrich), o-phosphoric acid (VWR), 1,4-diaminobutane (DAB) (99%, Aldrich), imidazole (ACS reagent, ≥99%, Sigma-Aldrich), citric-acid*H_2_O (ACS reagent, ≥99.9%, VWR), sodium chloride (99–100.5%, Sigma-Aldrich), phosphate buffer saline (PBS) (Tablet, Sigma), sodium hydroxide (VWR International, USA) were of analytical grade and were used as received. For the aqueous solutions, ultrapure water (Human Corporation ZeneerPower I Water Purification System) was used. For 1 L of imidazole buffer imidazole (pH 8: 12.988 g), citrate (pH 8: 1.728 g), sodium chloride (pH 8: 11.466 g) and ultrapure water were used. In all cases the exact pH was adjusted by the addition of hydrochloric acid and followed by digital pH meter (Thermo Scientific™ Orion™ 4-Star Plus pH/ISE Benchtop Multiparameter Meter).

For the *in vitro* experiments MG-63 human osteosarcoma cell line (Sigma-Aldrich, USA), Minimum Essential Medium (Gibco, USA), fetal bovine serum (Gibco, USA), non-essential amino acids (Gibco, USA), L-glutamine (Gibco, USA), penicillin and streptomycin (Gibco, USA), commercially available cell proliferation reagent (WST-1, Roche, Switzerland), Vybrant DiD vital dye (Molecular Probes, USA) were used.

### 2.2. Preparation of polysuccinimide and cysteamine modified polysuccinimide

PSI was prepared by thermal polycondensation of L-aspartic acid in the presence of o-phosporic acid catalyst, under vacuum at 180°C as described in our previous paper (Fig 1A) [18]. For the modification of PSI with cysteamine (CYSE), first 0.04 g of CYSE was dissolved in a mixture of 0.7 g DMF and 0.4 g DMSO in a glass reactor, then 2 g of PSI dissolved in DMF (25 w/w%) was added and mixed vigorously under Argon atmosphere at room temperature for 45 minutes (Fig 1C). After the synthesis, the final polymer concentration was 15 w/w% and theoretically, every 10^th^ repeating unit was modified with cysteamine (PSICYSE). Details of the synthesis and chemical characterization can be found in our previous paper.^29^

### 2.3. Preparation of electrospun cystamin crosslinked polysuccinimide fibers

Cystamine crosslinked PSI fibers were prepared by a homemade apparatus consisting of a Genvolt 73030P high voltage power supply, a KD Scientific KDS100 syringe pump, a Fortuna Optima 7.140 33 glass syringe with Luer-lock, a blunt G18 needle made by Hamilton and a grounded plate collector covered with aluminum foil. The electrospinning parameters were set at 0.8 ml/h flow rate, 15 cm target distance (distance between the tip of the needle and collector) and 12 kV potential. Samples were prepared from 1 ml of 15 w/w% PSICYSE solution. During the electrospinning process, as fibers were expelled from the needle, the thiol side chains of the PSICYSE formed disulfide bonds between the polymer chains upon oxidation by the atmospheric oxygen before reaching the collector (Fig 1D). The crosslinked polymer is denoted as PSICYS. Due to practical reasons and restrictions of our current setup the amount of electrospun material we can synthesize during a single electrospining session is limited. A simple solution to this issue is removing a sample from the aluminum collector, folding it then compressing it resulting in smaller yet thicker membranes. In our case 3 x 4 cm rectangles were prepared (Fig 1G). To reinforce them, they were then compressed by 5 t weight along their whole surface for 5 min using an Atlas Manual 15T Hydraulic Press (GS15011). Finally, disks of 16 mm diameter (Fig 1G) were cut out and subsequently sterilized by dry heat thermal sterilization in a Memmert SLP 500 at 120 ^o^C for 2 hours [32]. According to thermal gravimetry and differential thermal analysis, there is no physical or chemical change in the samples after applying this sterilization method: further details can be found in our previous paper [26].

### 2.4. Preparation of 1,4-diaminobuthane crosslinked PSI fibers (PSIDAB)

25 w/w% PSI/DMF solution was electrospun at 15 cm collector distance and 1 ml/h feeding rate (Fig 1E). 1 x 1 cm square samples were then cut and immersed in a 0.5 M DAB/EtOH solution (crosslinker solution) for different time intervals (1 min, 5 min, 10 min, 20 min, 30 min, 60 min, 120 min, 180 min, 1 day). After the reaction, samples were thoroughly immersed in DMF as a simple dissolution test. The reaction between PSI and DAB can be seen on Fig 1F. For the *in vivo* experiments, the PSIDAB samples of the 1-hour crosslinking time were chosen. Before the crosslinking, (similarly to the PSICYS samples) the PSI membrane was folded and compressed, then disks of 16 mm diameter were cut before immersing them in the crosslinker solution. Finally, samples were thoroughly washed with ultrapure water. Samples were securely sealed and stored in ultrapure water containing a small amount of ClO_2_ for sterilization (Fig 1F) [33].

### 2.5. Characterization

#### 2.5.1. Scanning Electron Microscopy (SEM)

For SEM studies, the samples were treated in different ways according to their type: PSI-based membranes: a small part of the membrane was cut out and placed on conductive tape for coating and microscopy; PASP-based membranes: samples were first thoroughly washed with ultrapure water and freeze-dried, then a small portion was placed on conductive tape for coating and microscopy; samples from *in vivo* experiments: a small portion of membranes was resected from the *in vivo* samples and was washed in an excessive amount of 100 mM Na-cacodylate (pH 7.2) solution, then stored in a 1 V/V% glutaraldehyde solution in 100 mM Na- cacodylate (pH 7.2). To dehydrate the membranes, the samples were first placed for 5 min in a series of ethanol solutions: 20, 50, 70, 85, 96 V/V% (diluted with water) then in a 1:1 ethanol (96 V/V%) and acetone mixture and finally in a porous container with pure acetone. In a slow process, acetone was replaced with supercritical CO_2_ and slowly heated until complete evaporation. The dry samples were then placed on conductive tape for coating and microscopy. Micrographs were taken using a ZEISS EVO 40 XVP scanning electron microscope equipped with an Oxford INCA X-ray spectrometer (EDS). An accelerating voltage of 20 kV was applied. For the measurements, samples were sputter-coated with gold in 20–30 nm thickness with a 2SPI Sputter Coating System. Fiber diameters were measured with ImageJ software. In every case where membranes had clear fiber morphology, 50 individual fibers were measured and averages were calculated.

#### 2.5.2. Multiphoton microscopy

Multiphoton microscopy enables the in-depth investigation of samples that either have auto-fluorescent properties or have been labelled with fluorescent dyes prior to the investigation. For the examination of PSI and PASP based membranes, a two-photon microscope (Femto2d, Femtonics, Hungary) with a Spectra Physics Deep See laser was used at an 800 nm wavelength to induce the auto-fluorescence of PSI and PASP [25]. The emitted photons were detected in the green channel. Images were taken with 10x objective by the MES4.4v program. The brightness and contrast of the pictures were enhanced for better visualization.

#### 2.5.3. Attenuated Total Reflectance Fourier Transform Infrared Spectroscopy (ATR-FTIR)

Chemical structures and the success of synthesis were investigated with a Jasco FT/IR-4700 (Able-Jasco, Japan) with DTGS detector. All spectra were collected over the range 400–4000 cm^-1^ at a resolution of 2 cm^-1^. The background spectra were measured on a clean and dry diamond crystal. The number of scans accumulated was 128.

#### 2.5.4. Synthesis of PASP-based membranes by the hydrolysis of the PSI membranes

The hydrolytic stability of PSI and PASP based samples were investigated *in vitro*. PASP samples were prepared by mild alkaline hydrolysis of the electrospun PSI membranes (PSICYS or PSIDAB) in an imidazole-based buffer solution of pH 8 (I = 250mM). The chemical reaction of PSI turning into PASP is depicted in Fig 1B. A consequence of the hydrolysis is the swelling of the membranes [28]. 100% conversion is achieved when the membranes reach their equilibrium size. Based on our previous study, PSIDAB spheres with a diameter of 5.5 mm obtain their equilibrium size in 5 hours [28]. Furthermore, the smaller the spheres were, the faster they obtained their equilibrium size during hydrolysis. Although the fiber in the membranes are much smaller, to ensure complete hydrolysis samples were kept in the buffer for 24 hours. The swelling investigation was performed as it is crucial regarding technical surgical aspects but also to compare any *in vitro* and *in vivo* differences. The diameter of 3 PSI disks was measured before and after hydrolysis with a caliper. Changes in thickness were considered negligible therefore the degree of swelling was calculated as diameter PASPDAB/PSIDAB*100. The average fiber diameter was calculated with standard error (confidence of 95%) using standard procedure.

After the hydrolysis, samples were washed with ultrapure water to get rid of the salts and were subsequently freeze-dried for SEM and ATR-FTIR. PASP membranes are denoted by changing the PSI part in the original sample name to PASP (PASPCYS, PASPDAB).

#### 2.5.5. Mechanical analysis

Mechanical analysis of the PSICYS and PSIDAB membranes was performed to assess their loading capacity but also investigate whether they can be reliably used during a standard surgical procedure (suturability). Therefore, the methods we followed aimed to assess both the properties of the materials themselves, as well as the membrane-suture interactions. All samples were immersed in saline to replicate the *in vivo* environment where the samples would inevitably swell. For the measurements, a ZWICK Z005 tensile testing machine (Ulm, Germany) was used with standard clamps holding the samples, while registering the force and displacement of the crosshead at a constant pulling rate of 10 mm/min. The initial sample size was always 2 cm long and 1 cm wide. Sample thickness was measured with a caliper. From the recorded data, the maximum bearing load was obtained. The ultimate tensile strength was calculated by dividing the maximum bearing load by the initial cross-section area. From each sample type at least 4 parallel samples were measured. In the case of suture-sample interaction investigation (Suture Retention Test), on one side of the previously mentioned samples, a simple interrupted suture was placed in the center 0.5 cm from the top and 0.5 cm from each side. The suture was pulled out from the fixed sample at a constant speed of 10 mm/min. This is in line with the recommendation of Pensalfini *et al*. for the measurement of suture retention strength and is in line with AAMI/ISO/ANSI 7198 Standard (2016) [34]. In their work they found that sample width of 1 cm and suture placed 0.5 cm from the edge of the sample are necessary to exclude any effect of sample geometry on the suture retention.

### 2.6. *In vitro* tests

#### 2.6.1. Cell culture

A human osteosarcoma cell line, MG-63 was cultured as a subconfluent monolayer under standard conditions (100% humidity, 37°C and 5% CO_2_) in a humidified incubator (Nuaire, USA). These osteoblast-like cells were cultured in Minimum Essential Medium, supplemented with 10% fetal bovine serum, 1% non-essential amino acids, 2 mM L-glutamine, 100 units/ml penicillin and 100 mg/ml streptomycin.

#### 2.6.2. Cell viability assay

Disks of an average diameter of 6 mm were cut from the electrospun membranes. To minimize the probability of a bacterial or fungal infection, the samples were stored in sterile-filtered PBS containing sodium azide. Before introducing the disks to the cells, the membrane samples were sterilized in a 300 ppm chlorine-dioxide solution (in PBS) for 10 min and they were incubated in the completed medium for 1 hour. First, the gel disks were placed into the wells of *low cell binding* 96 well microplates (flat bottom, Nunc, Denmark). After that, the MG-63 cells were seeded onto the gel disks at a concentration of 20 000 cells/well in 200 μl medium/well and incubated for 24 or 72 hours at 37°C.

Cell viability was evaluated by a colorimetric assay using a commercially available cell proliferation reagent (WST-1). The WST-1 reagent was diluted with uncompleted MEM solution (lacking phenol red) in a ratio of 1:20. After washing the wells with PBS to remove the non-attached and slightly attached cells, 200 μl of WST-1 solution was added to each well and the cells were incubated at 37°C for 4 h. The absorbance of the supernatant was measured at 450 nm with a reference wavelength of 655 nm using a microplate reader (Model 3550, Bio-Rad Laboratories, Japan).

#### 2.6.3. Multiphoton microscopy of the cells

To visualize the MG-63 cells growing on the surface of the fibrous membranes, they were labelled with the fluorescent vital dye Vybrant DiD before seeding (according to the manufacturer’s suggested protocol). Membrane disks of 6 mm in diameter were placed into Lab-tek 8 chamber slides (Nunc, USA) with tissue culture surface treatment, and 40 000 cells were seeded onto each disk. After 24 hours, the samples were fixed in 4% paraformaldehyde (in PBS) at RT and they were stored in PBS at 4°C until investigation under a multiphoton microscope. The red fluorescence shows the cells due to the Vybrant DiD vital staining while the green color indicates the autofluorescence of the PSI or PASP based membranes [25].

### 2.7. Animal model

Biocompatibility and biodegradability of electrospun PSICYS and PSIDAB samples were investigated on 24 male Wistar rats (250 g) as 12 parallel measurements were carried out for each sample type. Before implantation, the PSICYS and PSIDAB samples were immersed in sterile physiological saline (0.9%) for 10 minutes to reach their equilibrium size. Sedation of animals was performed with a mixture of ketamine and xylazine (~0.8 ml/animal). Samples were implanted at the nape under the skin. After a 1–2 cm long incision along the dorsal midline, samples were placed and fixed on the paramedian line via a single simple interrupted suture using an Atramat 2-0 polyglycolic acid absorbable suture material. Skin closure was performed with 3–4 simple interrupted stitches using the same suture material. Post-operatively for both PSICYS and PSIDAB, animals were randomly divided into two groups of 6 animals each. Animals were kept in individual cages and observed daily for evidence of wound complications, such as skin dehiscence seroma, hematoma, or infection. The experimental protocol adhered to rules laid down by the Directive of the European Parliament and of the Council on the protection of animals used for scientific purposes and was approved by the Semmelweis University’s Institutional Animal Care and Use Committee. The accreditation number of the laboratory is 22.1/1244/3/2015. We have not used control animals in the experiments for two reasons: firstly, according to 2010/63/EU guideline of the European Union, in animal studies the number of animals used should be reduced as low as reasonably possible; if applicable animals should be replaced with animals of lower hierarchy or other types of studies (*in vitro*); additionally, the *in vivo* study should be refined to provide animal care of the highest possible standards. Secondly, for the control animal, the surgical procedure would just involve a simple incision on the skin of the animal then sutured with the same technique and material. This would result in a standard wound healing process that is comprehensively documented in basic pathology books, like Robbins Pathology [35]. Termination and sample retrieval were performed after 3 days (Group A) and 7 days (Group B). Samples were preserved in formaldehyde and then sent for histological evaluation, whereas in the case of PSI-DAB, SEM micrographs were also taken from retrieved and then freeze-dried samples.

#### 2.7.1. Histology

PSIDAB and PSICYS samples were collected on the 3rd and 7th days after implantation as follows: the skin tissue was separated, and the samples were dissected around the suture including the muscle tissue then placed in 4 V/V% formaldehyde solution. After fixation, water was eliminated according to a standardized protocol in a Leica ASP300 enclosed tissue processor, samples were then embedded in paraffin and slices of 4 μm thickness were cut (Leica microtome). After removing the paraffin, slices were stained by standard haematoxylin-eosin staining protocol. All slides were digitalized with a Pannoramic 250 Flash Scanner (3DHISTECH Ltd.). Although no standardized protocol for specifically evaluating foreign body inflammatory reactions exists, some indicators are widely accepted as criteria of grading the inflammatory response and used in other areas of medicine. For quantifying the immune response and inflammatory grade to the implanted samples, a scoring system based on the work of Planck *et al*. was used as shown in Table 1 [36]. A small piece from the 7^th^ day PSIDAB was collected with a tweezer and investigated by a multiphoton microscope without any staining, and after freeze-drying by SEM to try and visualize leukocytes (macrophages, lymphocytes).

**Table 1:** Scoring system used to evaluate the histopathology samples [35].

Point Value	0	1	2	3	4
**Average Score**	0–0.49	0.50–1.49	1.50–2.49	2.50–3.50	> 3.50
**Inflammation Grade**	None	Mild	Moderate	Severe	
**Macrophage infiltration**	None	Mild	Moderate	Severe	
**Lymphocyte Infiltration**	None	Mild	Moderate	Severe	
**Foreign Body Giant Cell Formation**	None	Mild	Moderate	Severe	
**Granulation Tissue and Fibrosis thickness**	None	Narrow Band	Moderate Band	Wide Band	Extensive Band

### 2.8. Statistical analysis

When applicable, the data written in the text is represented by an average followed by the standard error calculated at p = 0.05 confidence level. The same confidence level was used for all calculations. The number of elements in each sample is given in brackets at the corresponding averages and errors. The error bars on figures represent the standard errors. For the hypothesis tests in Section 3.5 double-sided, two-sample Student t-tests were conducted using the averages and standard deviations of the samples. In the *in vitro* analysis Kruskal-Wallis ANOVA and median test were used for statistical evaluation of the data, applying the STATISTICA 10 software (Statsoft, USA). In case of p < 0.05 was considered a difference as statistically significant.

## 3. Results and discussion

### 3.1. Preparation of PSI, PSICYS and PSIDAB membranes

**Fig 2:**
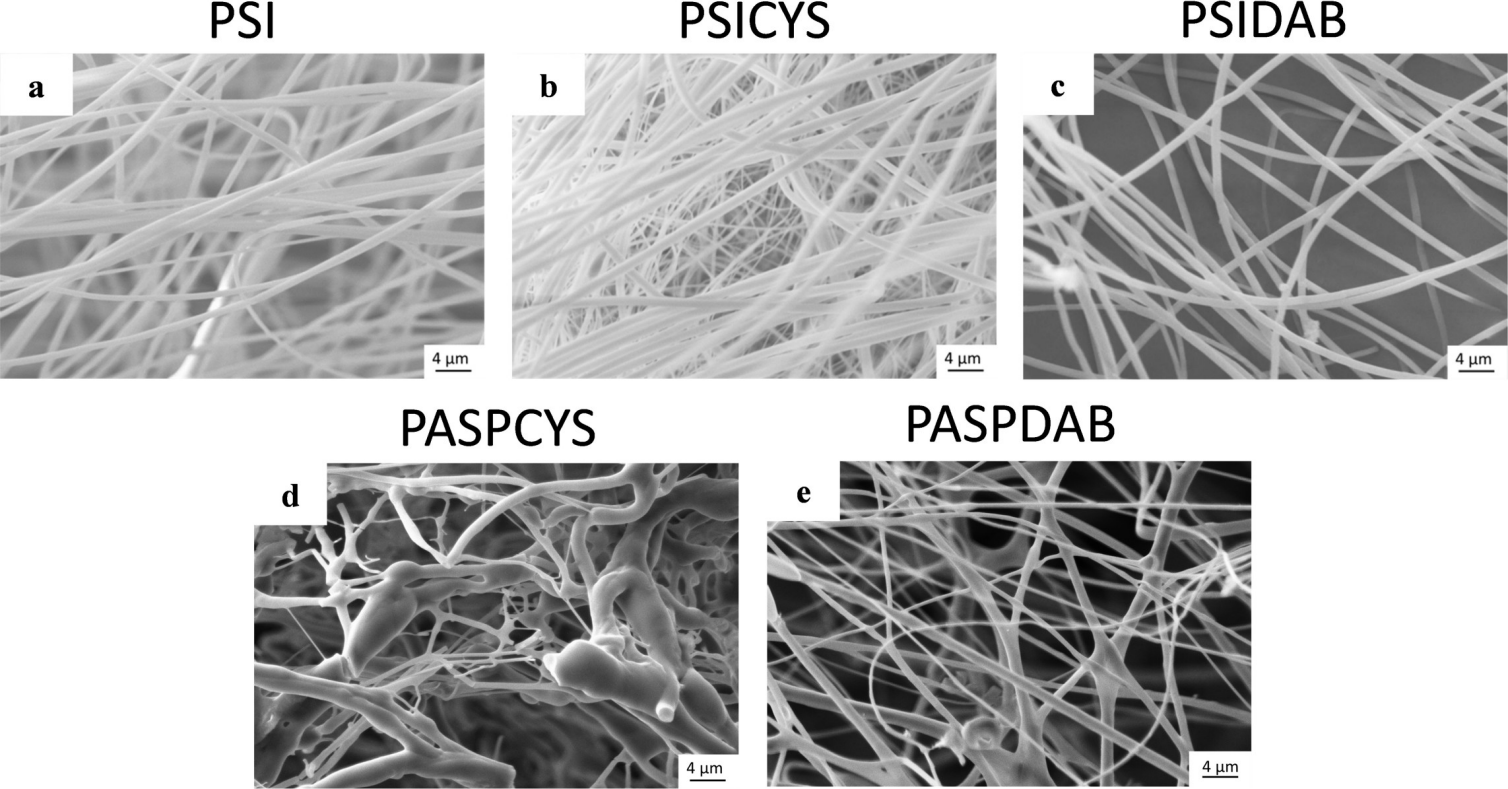
SEM micrographs of electrospun PSI fibers (a), PSICYS fibers (b), PASPCYS fibers (c), PSIDAB fibers without compression (d), SEM (e) and multiphoton micrographs (f) of freeze-dried PASPDAB fibers.

Regarding the synthesis, electrospinning and physicochemical properties of PSI and cysteamine modified polysuccinimide (PSICYSE) can be found in detail in our previous article.^29^ After electrospinning of both PSI and PSICYS a white sheet of fibrous material was obtained, that was easy to remove from the collector. Small parts of the white fibrous sheets were dipped in N,N-dymethilformamide (DMF) to check dissolution. The PSI sheet dissolved immediately contrary to the PSICYS sheet that only swelled indicating the presence of crosslinks. The reactive electrospinning had no significant effects on the fiber formation as the average diameter of PSICYS fibers (900 ± 70 nm (n = 50), Fig 2B) does not differ significantly from that of the PSI fibers (910 ± 80 nm (n = 50), Fig 2A). According to the SEM images, both the PSI and the PSICYS fibers had a smooth surface without any defects.

The idea for the implementation of post electrospinning crosslinking for the synthesis of PSIDAB was to immerse the fibrous membrane in the solution of a crosslinker to elicit crosslinks between the polymer chains inside the fibers. To check whether this hypothesis was right, previously prepared PSI fiber membranes were immersed in a 0.5 M DAB/EtOH solution for different periods and then washed with DMF to confirm dissolution. DAB is a feasible crosslinker for PSI as proven in some published works [18, 37]. PSI is soluble in N,N-dimethylformamide, dimethyl sulfoxide and partly in N-methylpirolidone. To prevent PSI from dissolving, we chose ethanol as the reaction solvent in which the crosslinking process is a heterogeneous reaction. After a 1- and 5-minute long immersion time in DAB/EtOH, samples dissolved in DMF. Only after a 10-minute immersion time did samples remain intact when immersed in DMF. In other words, the minimal reaction time for crosslinking of this system is 10 minutes. However, to ensure full crosslinking and reproducibility, 1 hour of crosslinking time was chosen at the preparation of PSIDAB for the *in vivo* experiments as no significant difference was found in either shape or morphology between the 10 minute and 1hour samples (S1 Fig in S1 File). The average fiber diameter of PSIDAB was slightly smaller (800 ± 30 nm, (n = 50)) (Fig 2C), compared to the PSI fibers used in this series (average fiber diameter was 911 ± 41 nm, (n = 50)), since the more crosslinks there are in a hydrogel, the more it shrinks.

### 3.2. Preparation of PASPCYS and PASPDAB membranes

Our objective was to synthesize fibrous samples that can be easily degraded *in vivo* (fast biodegradation) and samples whose degradation takes a longer time (slow biodegradation). PSICYS and PSIDAB differ from each other in both synthesis and chemical structure, which leads to different reactions *in vivo*. Disulfide bridges, such as the ones in PSICYS, are often in the focus of drug delivery research since those are cleavable in redox environments found *in vivo* [18, 37]. On the contrary, PSIDAB contains crosslinks that are not sensitive to such conditions and thus theoretically cannot be cleaved that easily, resulting in longer *in vivo* biodegradation times. Juriga *et al*. demonstrated that PASPCYS bulk hydrogels degrade in a *collagenase type I* solution but also in minimal essential media used for cell cultivation, which supports the original idea of the fast dissolution of PSICYS membranes and slow degradation of PSIDAB samples [25]. The membranes in the present project were implanted in the animals in PSI forms. However, for biodegradability, the membranes must first undergo hydrolysis after implantation, in other words turn into their respective PASP forms (S2 and S3 Figs in S1 File). Without hydrolysis the biodegradation would be compromised. Typically, pH in the connective tissue is approximately 7.4 while in the skin ranges between 4–6. However, in the case of acute inflammation due to surgery or damage to the skin tissue, it can rise to pH 7.4 or in the case of chronic inflammation even pH 8 [38]. Since PSI hydrolyzes to PASP at pH≥7.4, it is strongly suggested that hydrolysis occurs *in vivo* for both PSICYS and PSIDAB samples implanted under the skin.

**Fig 3:**
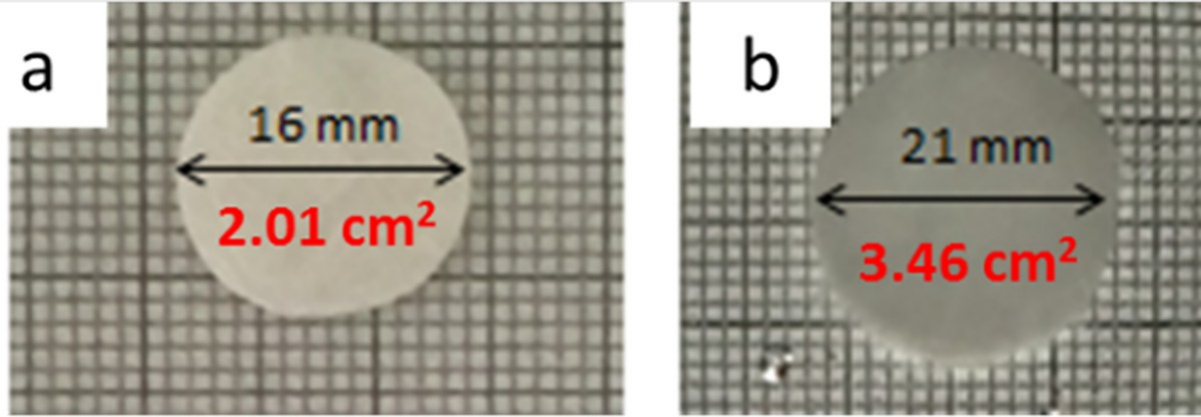
PSIDAB fibrous membrane before (a) and after hydrolysis (b), where red numbers are the area of samples.

As samples were implanted in PSI based form, it was essential to investigate morphological changes caused by hydrolysis. To imitate these, PSICYS and PSIDAB samples were hydrolyzed *in vitro* as well. During this process, the PSI based membranes turn into the PASP-based membranes and swell in aqueous medium. Considerable change was observed in the fibrous structure of PSICYS after hydrolysis. During hydrolysis, the PASPCYS fibers fuse creating bundles and bulk parts along the fibers, therefore the fibrous structure was only partially maintained (Fig 2D). This is often observable in gel fiber systems in different degrees. In the case of PSIDAB fibers, although crosslinking by itself did not change the fiber morphology, hydrolysis did. As evidenced by SEM images on washed and freeze-dried PASPDAB samples (with 1 hour crosslinking time), the fusion of fibers occurred in two ways: a. in some cases where fibers happened to be parallel and touched each other they fused into flat sheets (Fig 2E) b. in other instances where fibers touched each other in any degree an interconnected fibrous structure was created at the connection points. The fusion of fibers is not an unprecedented phenomenon and it has been observed in crosslinked networks [39, 40]. Zhang *et al*. reported a similar method for the post electrospinning crosslinking of PSI fibers with 1,2-diaminoethane in methanol solution, where the fibrous structure was severely damaged due to hydrolysis [41]. Our work demonstrates that PSI fibers crosslinked with DAB proved to be a better option for retaining the fibrous structure of PASPDAB. It caused minimal change in the fiber morphology, which alterations should not have any effect on either the mechanical or *in vivo* performance of the membranes. An example for the macroscopic changes of a PSIDAB–PASPDAB transition can be seen in Fig 3. In this case, the fibrous membranes grew from 16 ± 0 mm to 21 ± 1 mm, which corresponds to 31 ± 7% (n = 3, p = 0.05). Similar behavior was demonstrated for PSI based bulk hydrogels [42] but also other PASP based fibrous membranes with different crosslinkers as well [27]. Further details on the swelling behavior of different size PSIDAB membranes during hydrolysis and PASPDAB membranes in different pH solutions can be found in the supporting document. The detailed chemical analysis of PSI, PSICYS, PASPCYS, PSIDAB and PASPDAB can be found in the supplementary information and in S4 Fig in S1 File.

### 3.3. Mechanical analysis

Assessment of the mechanical properties of an implant is crucial before commencing *in vivo* experiments. While different methods are available to measure the stiffness, we chose uniaxial stress-strain measurements so we could obtain real-world practical results about the membranes that could be compared with *in vivo* tissues or market available implants and biomaterials [43]. Measurements were performed on wet PSI, PSICYS and PSIDAB membranes (all of the membranes were immersed in saline solution to mimic the environment of the *in vivo* experiments). In this regard, tensile strength is generally documented in one of two units: a. In Force/area (N/m^2^) for tissue or materials with well-defined dimensions or b. In Force/length (N/cm) for more complex materials with hard to define dimensions e.g., surgical meshes and specialized dressings. We present results as applicable.

**Fig 4:**
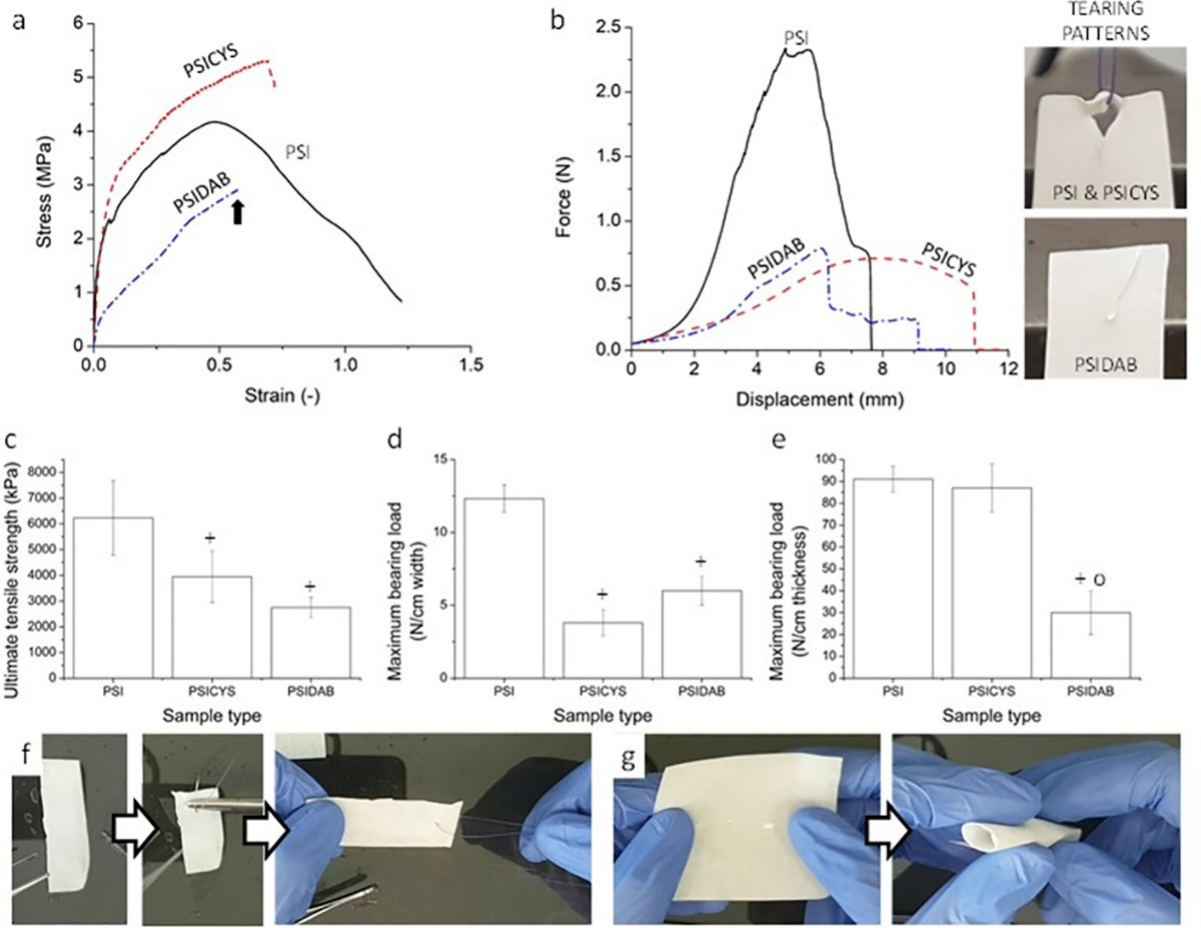
Examples for stress-strain curves of PSI, PSICYS and PSIDAB samples (black arrow indicates the tearing point of PSIDAB) (a), examples for force-displacement curves obtained in single interrupted suture model tests (b); ultimate tensile strengths (c), maximum bearing loads (d, e) of the samples; sutured PSIDAB sample (f), rolled up PSIDAB sample for e.g., laparoscopy (g). “+” means significant (p = 0.05) from PSI and “o” means significant difference from PSICYS.

In the first set of experiments, the maximum load-bearing capacity and the ultimate tensile strength of the raw membranes were investigated (Fig 4A and S5 Fig in S1 File). All samples exhibited similar initial behavior as their stress-strain curves are very steep, representing the high rigidity of the materials. The ultimate tensile strength of the membranes PSI was 6230 ± 1450 kPa or 12.32 ± 0.94 N/cm (n = 8, p = 0.05) of PSICYS it was 3947 ± 1001 kPa or 3.8 ± 0.9 N/cm (n = 5, p = 0.05) and of PSIDAB it was 2749 ± 390 kPa or 6.0 ± 1.0 N/cm (n = 6, p = 0.05) (Fig 4C and 4D). Since the standard errors in the measurements were quite high, we used two-sample t-test to see if there was a significant difference between the membrane types. According to the analysis, there was no significant difference between the maximum bearing loads of PSICYS and PSIDAB, however, they were both significantly different from the PSI membranes. In terms of performance the membranes are not suitable as bone, cartilage, or tendon implants or surgical meshes for hernia repair as their tensile strength is simply not high enough. Nevertheless, they are suitable as soft tissue implants. The tensile strength of the membranes is comparable to the tensile strengths of soft connective tissues and visceral tissues [43–45].

In the second set of experiments, a simple interrupted suture was placed on the samples (Fig 4F), which was then torn out by the mechanical tester while the force and displacement of the crosshead were registered (Suture Retention Test). Similarly, the assessed properties were the ultimate tensile strength and the maximum load-bearing capacity. Typical force-displacement curves of PSI, PSICYS and PSIDAB can be seen in Fig 4B whereas all the measured curves can be found in S6 Fig in S1 File.

In every case, the maximum bearing load of the suture retention test was considerably smaller than compared to the raw sample measurements. Ultimate tensile strength would be in theory calculated by dividing the maximum sustained load with the thickness of the suture material. However, the results would be highly overestimating the strength of the membranes. A relative tensile strength was calculated by dividing the maximum bearing loads by the thicknesses of the samples. For PSI membranes it was 91 ± 6 N/cm (n = 4, p = 0.05), for PSICYS membranes it was 87 ± 11 N/cm (n = 6, p = 0.05) while for PSIDAB membranes it was 30 ± 10 N/cm (n = 6, p = 0.05) (Fig 4E). According to two-sample t-tests, the PSIDAB once again was significantly different from the PSI and PSICYS samples that exhibited the same behavior. The load-bearing capacity of the sample may seem exceedingly small, but it is worth mentioning that no standardized criteria regarding suture retention tests are available. The measurement was rather performed to assess whether the membranes would withstand the surgical handling and *in vivo* suture fixation. Apart from instrument-based evaluations, during a manual surgical maneuver and suture fixation evaluation it was apparent (Fig 4G) that the membranes will indeed resist the intervention and fixation in the animals. Additionally, it is noteworthy that increasing the thickness of the fabricated membranes, will consequently result in an increase of their tensile strength as well. On the contrary, bulk hydrogels will still tear under the effect of sutures as their susceptibility to suturing is not in correlation with their thickness but with their texture.

### 3.4. *In vitro* experiments

Initially, both PASPCYS, PSIDAB and PASPDAB membranes were planned to be investigated. Although PASPCYS was stable in PBS for several days, it slowly degraded and lost its integrity completely during 8 days in the MEM solution (S7 Fig in S1 File). In contrast to this, PASPDAB could maintain its physical properties. This is in line with the expected biodegradability of the two materials. PSIDAB underwent hydrolysis and turned into PASPDAB, while shifting the pH of MEM toward the acidic region indicated by the MEM turning yellow.

**Fig 5:**
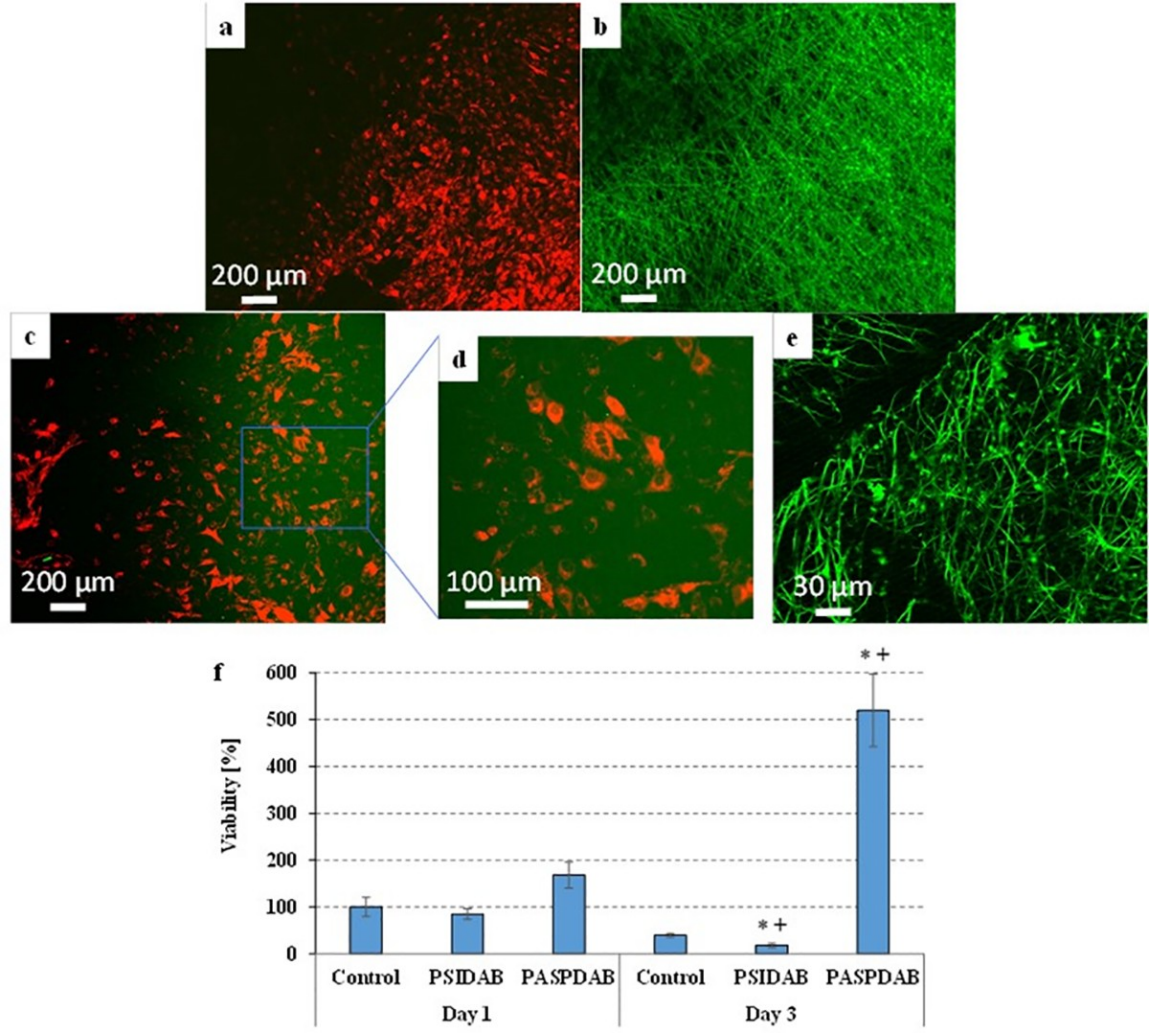
Multiphoton microscopic images of the Vybrant DiD-labelled MG-63 cells after 24-hour-long cultivation on the surfaces of ʺtissue cultureʺ plastic plates (a) or PSIDAB (b) or PASPDAB based (c and d) fibrous membranes and PASPDAB membrane without cells as a control (e). The cells show red fluorescence due to the Vybrant DiD staining while the green color indicates the autofluorescence of the PSI or PASP membranes. Red and green channels are both shown on all pictures except the (e) where only the green was active. Viability of MG-63 cells after 1- or 3-day-long cultivation on the surfaces of ʺlow cell bindingʺ plastic plates (control) and PSIDAB or PASPDAB membranes (f). * p < 0.05 compared to the control. + p < 0.05 compared to day 1.

By applying fluorescent pre-labelling, the cells could be visualized by multiphoton microscopy **(**Fig 5). On the plastic surface of tissue culture wells (Fig 5A), many healthy cells showing normal, star-like morphology can be seen. However, we could not find any cells on the PSIDAB membranes (Fig 5B), supposedly in this case the cells were only able to loosely attach to the membranes and all of them were removed during the fixation process including several washing steps. In addition, PSI based membranes shifted the pH value of the cell culture medium into the acidic range (its color always turned to yellow) indicating that these membranes do not support the survival of the cells under *in vitro* conditions. Nevertheless, large number of cells with healthy star-like morphology were observed on the PASPDAB membranes (Fig 5C and 5D). Fig 5E shows the control PASPDAB sample to demonstrate how the fibrous structure would look like without the cells.

The results of the viability assays **(**Fig 5F) show that the viability of MG-63 cells seeded onto PSIDAB and PASPDAB were similar to the control 1 day after the seeding. PASPDAB membranes showed no signs of cytotoxicity as the MG-63 osteosarcoma cells were able to attach to their surface and proliferate. These results are in agreement with our previous study, where biocompatibility of PASPDAB based bulk hydrogels was proven using the same cell line [25]. On the contrary the viability on PSIDAB decreased by day 3 (Fig 5F**).** As it was mentioned earlier, the MEM turned yellow due to the hydrolysis of PSIDAB marking a shift in pH toward the acidic range. MG-63 cells are not viable in acidic MEM which possibly caused the cell death, and a documented low viability. Therefore, based on this experiment it cannot be decided whether PSIDAB is cytotoxic as cell death most probably was caused by the pH shift. Nevertheless, this phenomenon is amplified in an *in vitro* setting as no fluid exchange is available. In contrast, in an *in vivo* environment, the body can readily compensate the lower pH. In this regard the results of the *in vivo* investigation should give further insight whether the shift is pH tolerable and whether meshes are biocompatible.

### 3.5. *In vivo* experiment

The purpose of the animal experiments was to investigate the surgical applicability of PSICYS and PSIDAB based membranes as suturable implants as well as to evaluate biocompatibility and biodegradability. The most important results of the *in vivo* studies are summarized in Table 2. The intra-operative surgical handling was easy for both materials and implantation took place without any difficulties. Samples were very flexible, and they could be rolled up or twisted in various degrees therefore their application is not limited to open surgical procedures but could be easily used for laparoscopic interventions as well (Fig 4D). Fixation with sutures was successful without any considerable damage to the implant. During the fixation suturability of the membranes was excellent and neither the sutures were torn out, nor the membranes were damaged. During the 3-day (Group A) and 7-day (Group B) observation no visible irritations, animal misbehavior or other macroscopically observable complications were detected. The animals behaved just as they did before surgery: normal food intake and bowel movement, mobility, and behavior with the caretakers were observed.

**Table 2:** Collection of results of *in vivo* experiments, where at the biocompatibility rows the determined scores can be seen inside the brackets.

		PSICYS	PSIDAB
**IMPLANTATION**	*Handling and surgery*	Easy handling, good suturability, no difficulties	
**AFTER 3 DAY**	*Macroscopic results*	Hydrolysis and swelling by approximately 40% in size	
	*Biocompatibility*	Moderate acute inflammation (1.83)	Moderate acute inflammation (2.17)
		Narrow band of granulation tissue (1.33)	Narrow band of granulation tissue (1.00)
	*Biodegradation*	No observable degradation	
	*Tissue invasion*	No tissue invasion according to histopathology	No tissue invasion according to histopathology Attachment of cells to the surface of the samples
**AFTER 7 DAY**	*Macroscopic results*	Samples either not found or completely covered and incorporated by new tissue	Samples lost their integrity and strength
			Easy truncation by tweezers
	*Biocompatibility*	Mild acute inflammation (1.17)	Mild acute inflammation (1.33)
		Moderate band of granulation tissue (1.50)	Narrow band of granulation tissue (1.33)
	*Biodegradation*	Although degradation was observable macroscopically, histopathology showed the remnants of fibrous samples at implantation area	Degradation was not observable, however the loss of mechanical strength and integrity indicates it
	*Tissue invasion*	Not according to histopathology	Cells invaded the samples for several millimeters
			Incorporation of samples into the granulation tissue

#### 3.5.1. 3-day results

**Fig 6:**
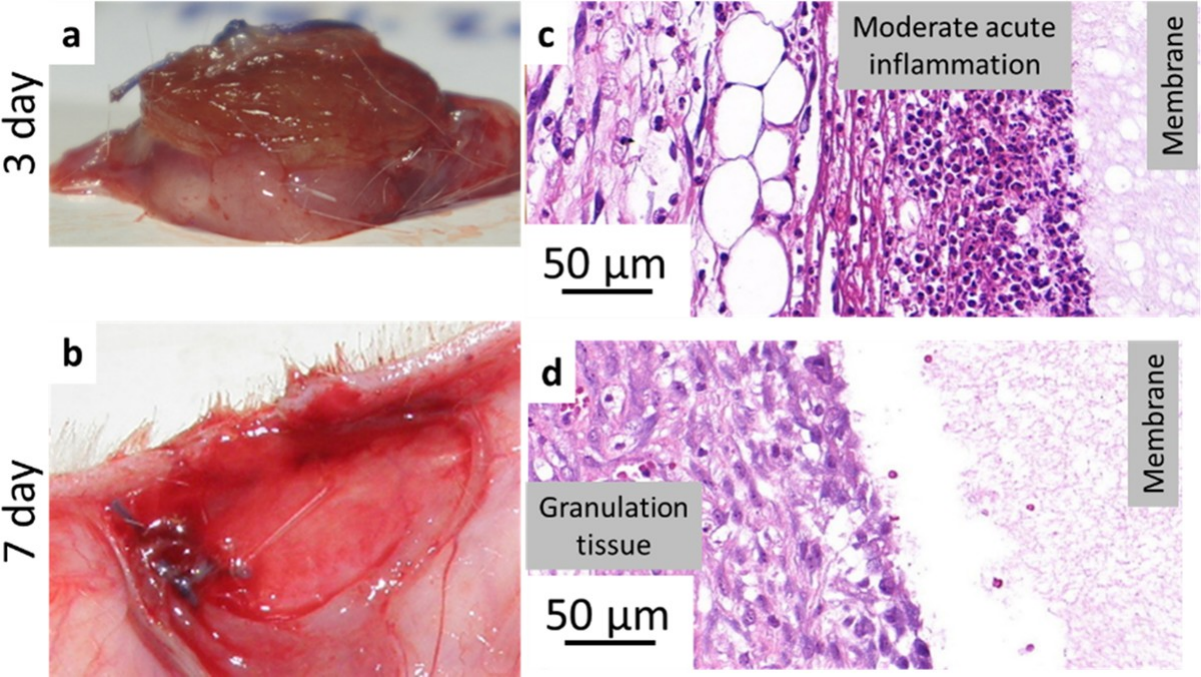
PASPCYS sample resected 3 days after implantation (a) and a representative image from histopathology (c); implantation area without sample after 7 days after implantation (b), a representative image from histopathology (d).

**Fig 7:**
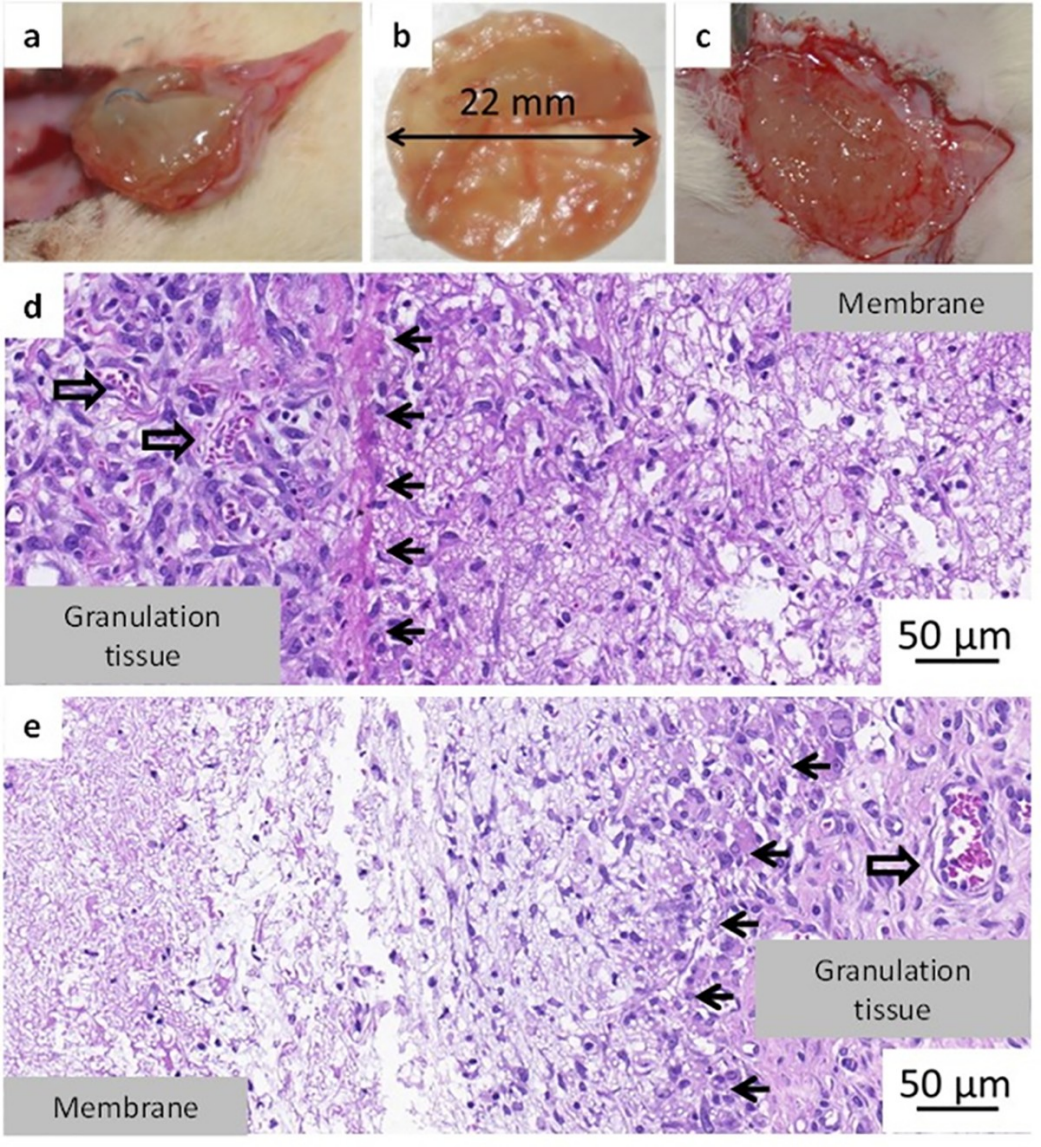
PASPDAB sample 3 days after implantation (a), resected sample grew in size and changed consistency (b), 7 days after implantation (c); representation of histopathology of a sample at day 3 (d) and day 7 (e); Black arrows indicate the boundary between the sample and the tissue and empty black arrows indicate blood vessels.

Three days after implantation (Group A) animals were terminated, implantation area was investigated, and the samples were resected. Both PSICYS (Fig 6A and S8 Fig in S1 File**)** and PSIDAB (Fig 7A) samples were found in their respective animals with physiological wound healing without any observable complications. There were no visible differences between the samples in different groups. However, both sample types grew by approximately 40% in diameter (from 16 mm to ~ 22 mm) and turned from the implanted white paper-like membranes to soft swollen gels (Figs 6A, 7A and 7B). Similar size change (16 mm to ~21 mm) due to hydrolysis was observed *in vitro* for both samples (Fig 3A and 3B, PSICYS not shown). This strongly suggests that hydrolysis of PSI based systems occurs *in vivo*, which is the first step in the biodegradation of PSI based materials. PSICYS and PSIDAB did not cause any irritation in the animals macroscopically nor created excessive skin tissue.

When a foreign object is implanted into a body, a natural response, the so-called foreign body type reaction is elicited [46]. Usually, it is a mild inflammation with granulocytes and histiocytes trying to restore the damaged area to its natural state for example by attacking or segregating the foreign object in a capsule (fibrosis) [47]. Although there is always some degree of foreign body type reaction and mild inflammation after any operation it is highly desirable to keep it minimal. Histopathological investigation as expected, revealed an acute inflammation in the tissue surrounding both types of samples further proving a physiological response of the animals to the implants. However, in neither case was this inflammation considered as a strong reaction. According to the scoring of *Planck et al*. [36] this inflammatory response is considered mild or moderate in the case of the PSIDAB samples (Table 2). PASPCYS samples were easily found on the slides surrounded by a rim of inflammatory cells predominantly comprising neutrophil granulocytes (Fig 6C and S9 Fig in S1 File), yet the cells did not invade the membrane. In the case of the PSIDAB samples the inflammatory reaction of the body was stronger, with a mixture of neutrophil granulocytes, lymphocytes and histiocytes surrounding and invading the outer part of the membrane, making it difficult to distinguish the tissue and the implant (Fig 7D and S10 Fig in S1 File, where the black arrows indicate the supposed boundary between the sample and the tissue). Around both membranes a narrow (Table 2). fibroblastic reaction and granulation tissue are visible (Figs 6 and 7), which will become new connective tissue. It is worth emphasizing, that no giant foreign body cells were observed in any of the samples for either membrane.

#### 3.5.2. 7-day results

Although similar results to the 3-day group were expected after 7 days group as well, significant differences were found between PSICYS and PSIDAB samples. In the case of PSICYS membranes (or in this state PASPCYS, because it was evidenced by the 3-day experiment that hydrolysis takes place) the samples were either not found in the animals (2 cases) or a soft new tissue was found in their place (4 cases) (Fig 6B). The soft granulation tissue grew around the suture, but it was easily deformed and removed. Upon cutting it half, there was no evidence for the presence of any PASPCYS remnants observable with the naked eye leading to the conclusion that these samples degraded and dissolved in 7 days. Furthermore, no signs of irritation, infection, or foreign body reaction neither on nor around the PASPCYS samples was found. Although macroscopically PASPCYS samples were impossible to find in the implantation area after 7 days, microscopically a fibrous substance (supposedly the PASPCYS matrix) was found with a moderate band of granulation tissue surrounding it (Fig 6D and S11 Fig in S1 File). In this state, the previously moderate inflammation declined to mild inflammation, while neovascularization (Table 2) was found in the granulation tissue. The new vessels formation (neovascularization) combined with the fact that no giant foreign body cells were found, highly indicates a healthy granulation tissue progressing to the proliferative phase of physiological wound healing.

**Fig 8:**
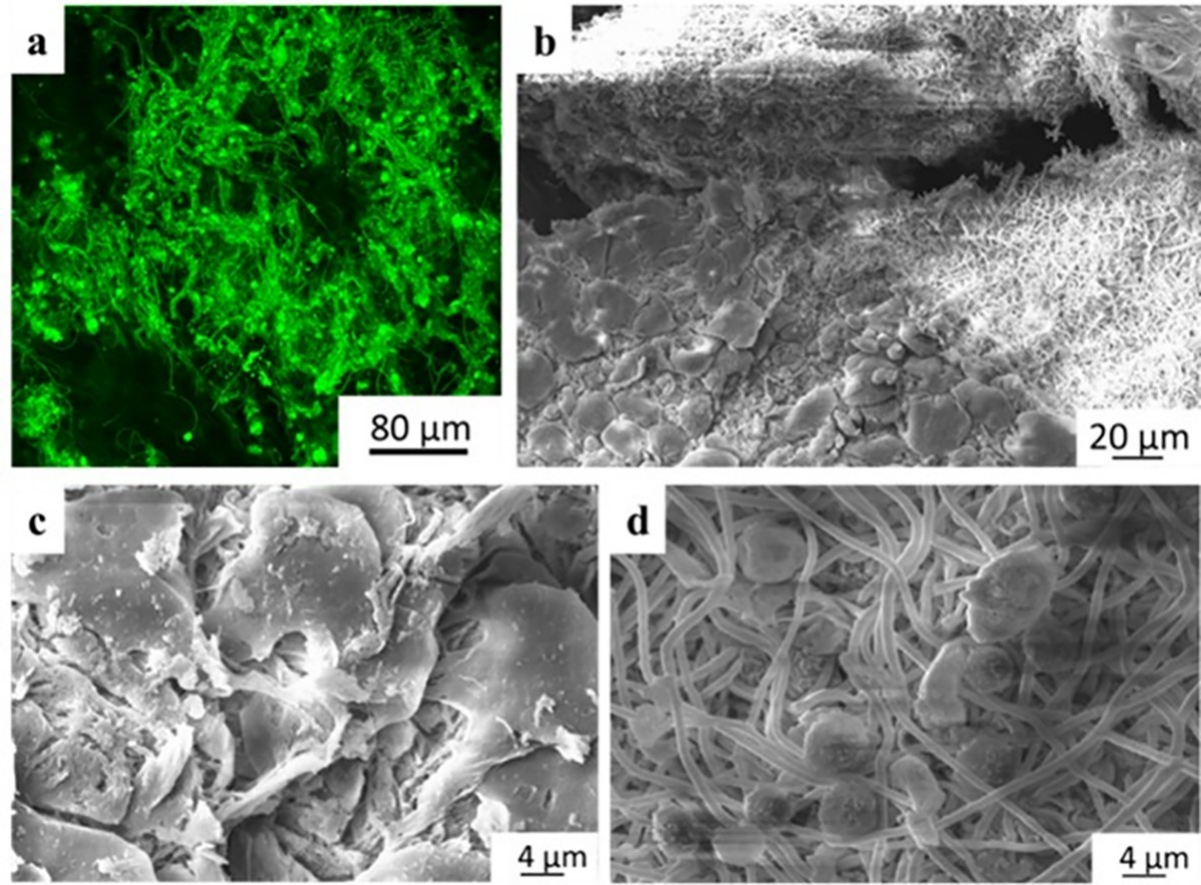
Multiphoton (a) and SEM (b, c, d) micrographs of PSIDAB sample from day 7.

In comparison, PASPDAB samples were found in a swollen, softer and more fragile state than their 3-day counterparts (Fig 7C). Therefore, hydrolysis has indeed occurred turning PSIDAB into PASPDAB. These samples were easily torn and dissected into separate layers or truncated by tweezers suggesting a high reduction in physical strength and consistency (S12 Fig in S1 File). Due to the deformation of the samples and incorporation of the surrounding tissue, it was hard to distinguish the samples from the granulation tissue macroscopically. Just as in the case of PSICYS, there were no macroscopic signs of irritation, infection, or foreign body type reaction neither on nor around the PASPDAB samples. Histopathology revealed the resolution of the acute inflammation observed on the 3^rd^ day post-implantation to a mild level (Table 2). Furthermore, the incorporation of the granulation tissue into the sample evolved into a stage where the boundary between the sample and granulation tissue was almost impossible to mark exactly (Fig 7E and S13 Fig in S1 File). The living tissue being able to invade the membrane is an exceptional result as it suggests a proper tissue integration. When examining biomaterials, a very frequent phenomenon is the encapsulation and segregation of the implant via a fibrous capsule which is not considered as true tissue integration [48]. Additionally, giant foreign body cells were not found in the granulation tissue surrounding these samples either, further supporting the results. We found that the granulation tissue band was still narrow even when not considering the part which grew into the samples. However, the thickness of granulation tissue surrounding the samples may have been just inaccurately determined. To determine the exact composition of the PSIDAB matrix after 7 days, a small sample was gathered with a tweezer and investigated by multiphoton- and scanning electron microscope without any treatment (Fig 8). Both the fibrous sample and the cells surrounding it were visible under the microscope glowing in green with similar intensities, and thus it was not possible to properly distinguish the two by digital subtraction. Nonetheless, the retained fibrous structure of the DAB crosslinked sample was still observable. As a reference, the PASPDAB fibrous microstructure can be found as prepared *in vitro* (without the cells) under the multiphoton microscope in Fig 5F.

This phenomenon was also observed on the scanning electron microscopy investigations of the PASPDAB samples resected 7 days post-implantation (Fig 8B and 8C). The depicted cells are impossible to recognize and classified as they were possibly flattened due to the preparation process [46]. It is also observable in the figure that under the cellular shell the fibrous structure was intact without any observable degradation or morphological change. This was also similar to the fibrous structure of the PASPDAB membranes prepared *in vitro* (Fig 5F). Furthermore, many spheres were found with 6–8 μm in diameter suggesting the presence of either granulocytes, lymphocytes or red blood cells inside the matrix close to the surface, which is understandable given that histopathology showed vascularization around these samples. Therefore, these are the most probable corpuscular elements (based on the literature) with comparable size with the ones found on the SEM images (Fig 8D) [49, 50].

When comparing the two membranes we can clearly see a difference in *in vivo* behavior. The shorter and longer biodegradation times of PSICYS and PSIDAB accordingly are evident. This verifies our hypothesis that both PSIDAB and PSICYS systems hydrolyze and swell *in vivo* turning into the corresponding PASP based systems. The exact degradation mechanism of PSICYS has yet to be clarified, while PSIDAB maintains its physical form after 7 days implanted under the skin showing reduction in physical strength and consistency, thus showing signs of degradation.

## 4. Conclusion

Poly(aspartic acid) (PASP) hydrogel systems possess advantageous properties such as biodegradability, biocompatibility and easy functionalization. In this work we have presented the fabrication and characterization of electrospun crosslinked fibrous polysuccinimide (PSI) membranes. These membranes can readily hydrolyze into PASP fibrous hydrogels. These provide a reliable system during the implantation and a template even closer to the body’s innate ECM. Two types of PSI fibrous membranes were prepared: one based on disulfide crosslinks exhibiting fast biodegradation and another based on alkyl-chain crosslinks showing longer biodegradation time. According to mechanical assessments, both membranes showed adequate mechanical properties for suturing and surgery. *In vitro* tests showed that the disulfide crosslinked membrane dissolves in cell culture conditions in 8 days whereas the alkyl-chain crosslinked one was still stable. Furthermore, the membranes exhibited no cytotoxic side effects as MG-63 osteosarcoma cells could attach to their surface and freely proliferate. Implantability was tested *in vivo* on small animals. Membranes were implanted in Wistar rats, under the skin at the backside of the neck. The membranes changed into PASP based membranes in 3 days and after 7 days most of the disulfide crosslinked membranes disappeared, proving biodegradation. Histopathologic examinations in both cases showed only a mild to moderate acute inflammation, which diminished after 7 days. Moreover, excessive tissue invasion was also observed proving the biocompatibility and tissue integration capabilities of these systems. These results coupled with the easily modifiable chemical structure of the poly(amino acid) based system makes them ideal for advanced functionalized materials and platforms for biomedical research.

## Supporting information

S1 File Supporting text and figures. Pictures of macroscopic fibrous gel-membranes; additional SEM images, all measured curves of mechanical investigation which was not included in the text, additional macroscopic pictures, and histopathological slides from the *in vivo* experiment.
(DOCX)

S1 Graphical abstract (TIF)

## References

[1] Caló E, Khutoryanskiy V V. Biomedical applications of hydrogels: A review of patents and commercial products. Eur Polym J. 2015;65: 252–267. doi: 10.1016/j.eurpolymj.2014.11.024

[2] Yeo CK, Vikhe YS, Li P, Guo Z, Greenberg P, Duan H, et al. Hydrogel Effects Rapid Biofilm Debridement with ex situ Contact-Kill to Eliminate Multidrug Resistant Bacteria in vivo. ACS Appl Mater Interfaces. 2018;10: 20356–20367. doi: 10.1021/acsami.8b06262 29806938

[3] Zhu H, Yang X, Genin GM, Lu TJ, Xu F, Lin M. The relationship between thiol-acrylate photopolymerization kinetics and hydrogel mechanics: An improved model incorporating photobleaching and thiol-Michael addition. J Mech Behav Biomed Mater. 2018;88: 160–169. doi: 10.1016/j.jmbbm.2018.08.013 30173068 PMC6392438

[4] Dong Y, Jin G, Hong Y, Zhu H, Lu TJ, Xu F, et al. Engineering the Cell Microenvironment Using Novel Photoresponsive Hydrogels. ACS Appl Mater Interfaces. 2018;10: 12374–12389. doi: 10.1021/acsami.7b17751 29537822

[5] Yao M, Gao F, Xu R, Zhang J, Chen Y, Guan F. A dual-enzymatically cross-linked injectable gelatin hydrogel loaded with BMSC improves neurological function recovery of traumatic brain injury in rats. Biomater Sci. 2019. doi: 10.1039/c9bm00749k 31355388

[6] Feksa LR, Troian EA, Muller CD, Viegas F, Machado AB, Rech VC. Hydrogels for biomedical applications. Nanostructures Eng Cells, Tissues Organs From Des to Appl. 2018;64: 403–438. doi: 10.1016/B978-0-12-813665-2.00011–9

[7] Calvert P. Hydrogels for soft machines. Adv Mater. 2009;21: 743–756. doi: 10.1002/adma.200800534

[8] Xue J, Xie J, Liu W, Xia Y. Electrospun Nanofibers: New Concepts, Materials, and Applications. Acc Chem Res. 2017;50: 1976–1987. doi: 10.1021/acs.accounts.7b00218 28777535 PMC6589094

[9] Wang K, Hou W Da, Wang X, Han C, Vuletic I, Su N, et al. Overcoming foreign-body reaction through nanotopography: Biocompatibility and immunoisolation properties of a nanofibrous membrane. Biomaterials. 2016;102: 249–258. doi: 10.1016/j.biomaterials.2016.06.028 27344368

[10] Damanik FFR, Spadolini G, Rotmans J, Farè S, Moroni L. Biological activity of human mesenchymal stromal cells on polymeric electrospun scaffolds. Biomater Sci. 2019;7: 1088–1100. doi: 10.1039/c8bm00693h 30633255

[11] Alessandrino A, Fregnan F, Biagiotti M, Muratori L, Bassani GA, Ronchi G, et al. SilkBridge^TM^: a novel biomimetic and biocompatible silk-based nerve conduit. Biomater Sci. 2019. doi: 10.1039/C9BM00783K 31359013

[12] Fu GD, Xu LQ, Yao F, Zhang K, Wang XF, Zhu MF, et al. Smart Nanofibers from Combined Living Radical Polymerization, “Click Chemistry”, and Electrospinning. ACS Appl Mater Interfaces. 2009;1: 239–243. doi: 10.1021/am800143u 20353208

[13] Dhand C, Venkatesh M, Barathi VA, Harini S, Bairagi S, Goh Tze Leng E, et al. Bio-inspired crosslinking and matrix-drug interactions for advanced wound dressings with long-term antimicrobial activity. Biomaterials. 2017;138: 153–168. doi: 10.1016/j.biomaterials.2017.05.043 28578293

[14] Kim Y-J, Ebara M, Aoyagi T. Temperature-responsive electrospun nanofibers for ‘on–off’ switchable release of dextran. Sci Technol Adv Mater. 2012;13: 064203. doi: 10.1088/1468-6996/13/6/064203 27877530 PMC5099763

[15] Molnar K, Jozsa B, Barczikai D, Krisch E, Puskas JE, Jedlovszky-Hajdu A. Plasma treatment as an effective tool for crosslinking of electrospun fibers. J Mol Liq. 2020;303: 112628–112636. doi: 10.1016/j.molliq.2020.112628

[16] Zeng Jun, Hou Haoqing, Joachim H. Wendorff AG. Photo-Induced Solid-State Crosslinking of Electrospun Poly(vinyl alcohol) Fibers. Macromol Rapid Commun Rapid Commun. 2005;26: 1557–1562. doi: 10.1002/marc.200500545

[17] Theron JP, Knoetze JH, Sanderson RD, Hunter R, Mequanint K, Franz T, et al. Modification, crosslinking and reactive electrospinning of a thermoplastic medical polyurethane for vascular graft applications. Acta Biomater. 2010;6: 2434–47. doi: 10.1016/j.actbio.2010.01.013 20080215

[18] Zrinyi M, Gyenes T, Juriga D, Kim J-H. Volume change of double cross-linked poly(aspartic acid) hydrogels induced by cleavage of one of the crosslinks. Acta Biomater. 2013;9: 5122–31. doi: 10.1016/j.actbio.2012.08.046 22975627

[19] Thombre SM, Sarwade BD. Synthesis and biodegradability of polyaspartic acid: A critical review. J Macromol Sci—Pure Appl Chem. 2005;42 A: 1299–1315. doi: 10.1080/10601320500189604

[20] Lim S, Nguyen MP, Choi Y, Kim J, Kim D. Bioadhesive Nanoaggregates Based on Polyaspartamide- *g* -C18/DOPA for Wound Healing. Biomacromolecules. 2017; acs.biomac.7b00584. doi: 10.1021/acs.biomac.7b00584 28678473

[21] Di Meo C, Cilurzo F, Licciardi M, Scialabba C, Sabia R, Paolino D, et al. Polyaspartamide-Doxorubicin Conjugate as Potential Prodrug for Anticancer Therapy. Pharm Res. 2015;32: 1557–1569. doi: 10.1007/s11095-014-1557-2 25366547

[22] Sharma A, Kundu S, Reddy M A, Bajaj A, Srivastava A. Design and Engineering of Disulfide Crosslinked Nanocomplexes of Polyamide Polyelectrolytes: Stability under Biorelevant Conditions and Potent Cellular Internalization of Entrapped Model Peptide. Macromol Biosci. 2013;13: 927–937. doi: 10.1002/mabi.201300018 23696522

[23] Craparo EF, Porsio B, Sardo C, Giammona G, Cavallaro G. Pegylated Polyaspartamide-Polylactide-Based Nanoparticles Penetrating Cystic Fibrosis Artificial Mucus. Biomacromolecules. 2016;17: 767–777. doi: 10.1021/acs.biomac.5b01480 26866983

[24] Kim M, Shin SW, Lim CW, Kim J, Um SH, Kim D. Polyaspartamide-based graft copolymers encapsulating iron oxide nanoparticles for imaging and fluorescence labelling of immune cells. Biomater Sci. 2017. doi: 10.1039/c6bm00763e 27999834

[25] Juriga D, Nagy K, Jedlovszky-Hajdú A, Perczel-Kovách K, Chen YM, Varga G, et al. Biodegradation and Osteosarcoma Cell Cultivation on Poly(aspartic acid) Based Hydrogels. ACS Appl Mater Interfaces. 2016;8: 23463–23476. doi: 10.1021/acsami.6b06489 27541725

[26] Molnar K, Juriga D, Nagy PM, Sinko K, Jedlovszky-Hajdu A, Zrinyi M. Electrospun poly(aspartic acid) gel scaffolds for artificial extracellular matrix. Polym Int. 2014;63: 1608–1615. doi: 10.1002/pi.4720

[27] Molnar K, Jedlovszky-Hajdu A, Zrinyi M, Jiang S, Agarwal S. Poly(amino acid)-Based Gel Fibers with pH Responsivity by Coaxial Reactive Electrospinning. Macromol Rapid Commun. 2017;201700147: 1700147–1700151. doi: 10.1002/marc.201700147 28488377

[28] Varga Z, Molnár K, Torma V, Zrínyi M. Kinetics of volume change of poly(succinimide) gels during hydrolysis and swelling. Phys Chem Chem Phys. 2010;12: 12670–12675. doi: 10.1039/c0cp00527d 20730186

[29] Wang B, Jeon YS, Park HS, Kim YJ, Kim JH. Mussel-mimetic self-healing polyaspartamide derivative gel via boron-catechol interactions. Express Polym Lett. 2015;9: 799–808. doi: 10.3144/expresspolymlett.2015.75

[30] Sharma A, Srivastava A. Pronounced influence of pH, metal-ion and solvent isotope on the thermoresponse of synthetic amphiphilic polypeptides. Polym Chem. 2013; 5119–5128. doi: 10.1039/c3py00741c

[31] Németh C, Szabó D, Gyarmati B, Gerasimov A, Varfolomeev M, Abdullin T, et al. Effect of side groups on the properties of cationic polyaspartamides. Eur Polym J. 2017;93: 805–814. doi: 10.1016/j.ijpharm.2016.12.007 27931785

[32] Weyenberg W, Vermeire A, D’Haese E, Vanhaelewyn G, Kestelyn P, Callens F, et al. Effect of different sterilisation methods on the properties of bioadhesive powders and ocular minitablets, and clinical evaluation. Eur J Pharm Sci. 2004;23: 77–87. doi: 10.1016/j.ejps.2004.05.010 15324925

[33] Noszticzius Z, Wittmann M, Kály-Kullai K, Beregvári Z, Kiss I, Rosivall L, et al. Chlorine dioxide is a size-selective antimicrobial agent. PLoS One. 2013;8: 1–10. doi: 10.1371/journal.pone.0079157 24223899 PMC3818415

[34] Pensalfini M, Meneghello S, Lintas V, Bircher K, Ehret AE, Mazza E. The suture retention test, revisited and revised. J Mech Behav Biomed Mater. 2018;77: 711–717. doi: 10.1016/j.jmbbm.2017.08.021 28867371

[35] Kumar V, Abbas KA, Aster JC. Robbins Basic Pathology. 10th ed. Elsevier Health Sciences; 2017. Available: https://www.elsevier.com/books/robbins-basic-pathology/kumar/978-0-323-35317-5

[36] Schmitt VH, Planck CN. Histological and Immunohistological Evaluation of the Tissue Response of a New Barrier Material Based on D,L-Polylactide, Trimethylene Carbonate and Caprolactone to Prevent Peritoneal Adhesion Formation. J Tissue Sci Eng. 2014;05. doi: 10.4172/2157-7552.1000138

[37] Krisch E, Messager L, Gyarmati B, Ravaine V, Szilágyi A. Redox- and pH-Responsive Nanogels Based on Thiolated Poly(aspartic acid). Macromol Mater Eng. 2016;301: 260–266. doi: 10.1002/mame.201500119

[38] Jones EM, Cochrane CA, Percival SL. The Effect of pH on the Extracellular Matrix and Biofilms. Adv Wound Care. 2015;4: 431–439. doi: 10.1089/wound.2014.0538 26155386 PMC4486717

[39] Lu W, Ma M, Xu H, Zhang B, Cao X, Guo Y. Gelatin nanofibers prepared by spiral-electrospinning and cross-linked by vapor and liquid-phase glutaraldehyde. Mater Lett. 2015;140: 1–4. doi: 10.1016/j.matlet.2014.10.146

[40] Jeffries EM, Allen RA, Gao J, Pesce M, Wang Y. Highly elastic and suturable electrospun poly(glycerol sebacate) fibrous scaffolds. Acta Biomater. 2015;18: 30–39. doi: 10.1016/j.actbio.2015.02.005 25686558 PMC4395539

[41] Zhang C, Wu S, Qin X. Facile fabrication of novel pH-sensitive poly(aspartic acid) hydrogel by crosslinking nanofibers. Mater Lett. 2014;132: 393–396. doi: 10.1016/j.matlet.2014.06.031

[42] Gyenes T, Torma V, Gyarmati B, Zrínyi M. Synthesis and swelling properties of novel pH-sensitive poly(aspartic acid) gels. Acta Biomater. 2008;4: 733–44. doi: 10.1016/j.actbio.2007.12.004 18280800

[43] Guimarães CF, Gasperini L, Marques AP, Reis RL. The stiffness of living tissues and its implications for tissue engineering. Nat Rev Mater. 2020;5: 351–370. doi: 10.1038/s41578-019-0169-1

[44] Colley H, McArthur SL, Stolzing A, Scutt A. Culture on fibrin matrices maintains the colony-forming capacity and osteoblastic differentiation of mesenchymal stem cells. Biomed Mater. 2012;7: 045015. doi: 10.1088/1748-6041/7/4/045015 22689305

[45] Todros S, Pavan PG, Pachera P, Natali AN. Synthetic surgical meshes used in abdominal wall surgery: Part II-Biomechanical aspects. J Biomed Mater Res Part B Appl Biomater. 2017;105: 892–903. doi: 10.1002/jbm.b.33584 26687728

[46] Daniell H, Anderson JM, Rodriguez A, Chang DT. Foreign Body Reaction to Biomaterials. Semin Immunol. 2008;20: 86–100. doi: 10.1016/j.smim.2007.11.004 18162407 PMC2327202

[47] Jones KS. Effects of biomaterial-induced inflammation on fibrosis and rejection. Semin Immunol. 2008;20: 130–136. doi: 10.1016/j.smim.2007.11.005 18191409

[48] Chandorkar Y, K R, Basu B. The Foreign Body Response Demystified. ACS Biomater Sci Eng. 2019;5: 19–44. doi: 10.1021/acsbiomaterials.8b00252 33405858

[49] Kim J, Nafiujjaman M, Nurunnabi M, Lim S, Lee Y-K, Park H-K. Effects of polymer-coated boron nitrides with increased hemorheological compatibility on human erythrocytes and blood coagulation. Clin Hemorheol Microcirc. 2018; 1–16. doi: 10.3233/CH-170307 29710679

[50] De Almeida HL, Coelho Bicca EDB, De Andrade MM, Andrade Neto PDR. Scanning electron microscopy of granuloma annulare. An Bras Dermatol. 2018;93: 740–742. doi: 10.1590/abd1806-4841.20187409 30156630 PMC6106657

