# Supplementary material for: Poly(amino acid) based fibrous membranes with tuneable in vivo biodegradation
Source: PLoS One. 2021 Aug 13;16(8):e0254843. doi: 10.1371/journal.pone.0254843 (PMC8362958; doi:10.1371/journal.pone.0254843)
Supplement: S1 File — Pictures of macroscopic fibrous gel-membranes; additional SEM images, all measured curves of mechanical investigation which was not included in the text, additional macroscopic pictures, and histopathological slides from the in vivo experiment. (DOCX) [file pone.0254843.s001.docx]

**poly(amino acid) based fibrous Membranes with tuneable IN VIVO biodegradation**

Kristof Molnar^1,2^, Constantinos Voniatis^1,3^, Daniella Feher^3^, Rita Varga^1^, Lilla Reiniger^4^, David Juriga^1^, Zoltan Kiss^5,6^, Eniko Krisch^2^, Gyorgy Weber^3^, Andrea Ferencz^3^, Gabor Varga^7^, Miklos Zrinyi^1^, Krisztina S. Nagy^1^, Angela Jedlovszky-Hajdu^1*^

^1^ Laboratory of Nanochemistry, Department of Biophysics and Radiation Biology, Semmelweis University, Nagyvárad tér 4, H-1089 Budapest, Hungary

^2^ Department of Food, Agricultural and Biological Engineering, College of Food, Agricultural, and Environmental Sciences, The Ohio State University, 222 FABE, 1680 Madison Avenue, Wooster, OH 44691

^3^ Department of Surgical Research and Techniques, Budapest, Semmelweis University, Budapest, Hungary

^4^1st Department of Pathology and Experimental Cancer Research, Semmelweis University, Budapest, Hungary

^5^ Department of Polymer Engineering, Faculty of Mechanical Engineering, Budapest University of Technology and Economics, Budapest, Hungary

^6^Biomechanical Research Centre, Faculty of Mechanical Engineering, Budapest University of Technology and Economics, Budapest, Hungary

^7^ Department of Oral Biology, Semmelweis University, Budapest, Hungary

*Corresponding author: Angela Jedlovszky-Hajdu, [hajdu.angela@med.semmelweis-univ.hu](file:///D:\Munka\cikkek\Írás%20alatt\PSI%20biokompatibilitás\ACS%20applied%20mat%20int\Resubmission\hajdu.angela@med.semmelweis-univ.hu)





**S1 Fig. PASPDAB samples with 10 minutes and 1 hour cross-linking times.**

**Swelling properties of PSIDAB and PASPDAB membranes**

pH responsive hydrogels, that exhibit volume change upon alterations of environmental pH are often in the focus of research in drug administration. As PASP exhibits a pH sensitive character due to carboxylic groups on the polymer’s repeating units, it can be also used for such applications as demonstrated previously both in bulk macroscopical [1] and nano systems [2]. Since PASPCYS showed very similar pH responsivity to PASPDAB, only the latter is discussed. The pH responsivity of PASPDAB was tested using disks of 3 different initial sizes (S2a, S2d and S2g Figs). PASPDAB samples swelled in pH 8 imidazole-based buffer and shrunk in pH 3 citric acid-based buffer solutions respectively. The deswelling and swelling of PASPDAB membranes happened in less than 1 minute. Due to the moving of the membrane after the buffer change, measurement of the kinetics of swelling and deswelling was not feasible. Therefore, only the equilibrium sizes after the swelling and deswelling finished, were measured. The average area of the disks was calculated. Results indicate that the swelling and de-swelling was consistent and reproducible throughout at least, 4 cycles of buffer changes (S3a Fig). A similar behavior was demonstrated by other PASP based fibrous membranes with different cross-linkers as well [3]. While the described pH responsivity of PASPDAB membranes is in line with the behavior of PASP based bulk hydrogels [4] there was one aspect where a difference was found. Swelling ratios of the disks are collected on S3b Fig, where swelling ratio means the size of the disks in any given state divided by its size in the first pH 8 solution in completely swollen state. These suggest that the bigger the average initial size of a disk is, the smaller the average relative size change. In other words, the bigger a disk is, the smaller the change in size between the swollen and the de-swollen states is. In order to investigate this hypothesis, a simple statistical analysis was carried out: the individual data points of S3b Fig were plotted as a function of initial dry diameter in S3c Fig. Then, linear fits were prepared on the pH 3 and pH 8 datasets. To assess whether a significant dependence of relative swelling degree on the size of the gel membrane exists, a double sided, one sample Student *t*-test was carried out using the slopes of the fitted lines. Our null hypothesis was that the slopes are 0. In both cases the calculated *t* parameters were outside of the acceptable range at 95 % confidence level, thus in both cases the null hypothesis was rejected. Hence, according to our data, the relative swelling degree significantly depends on the initial diameter of the gel sample. On the contrary, the swelling degree of a bulk macroscopic hydrogel of the same composition is independent of the initial size [5,6]. It is also important to note that, the larger the gel disk is, the more time it requires to reach its equilibrium size during swelling and de-swelling [6,7] and thus, the only possible explanations to why the fibrous membranes behaved differently compared to the bulk gel disks, is the structural difference between bulk and fibrous gels. Similarly, the smaller a hydrogel is, the faster it reaches its equilibrium size and in the case of the fibrous PASPDAB hydrogel fiber sizes used in the experiment, 24 hours was more than enough to reach equilibrium [6]. Evidently, the only possible explanation is that a gel disk composed of fibers swells slightly differently compared to bulk hydrogels.


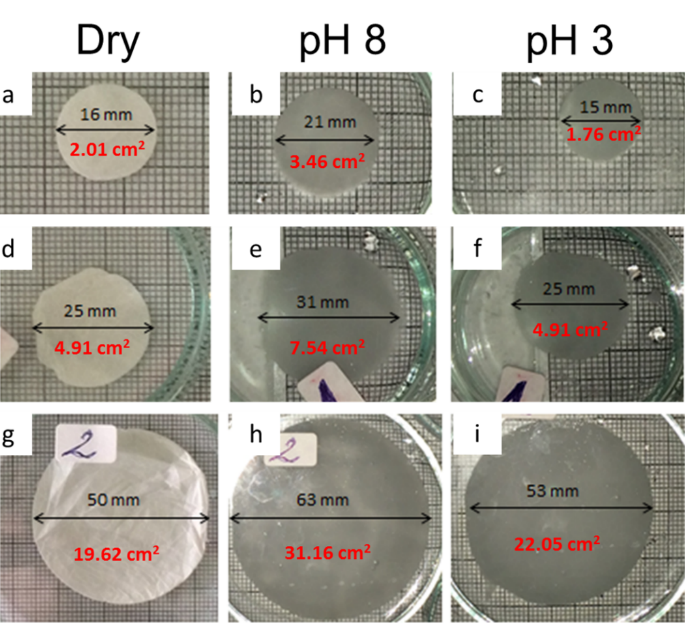


**S2 Fig. Dry PSI fibrous membranes of different sizes (a, b, c), same membranes after cross-linking with DAB in pH 8 buffer solution (b, e, h respectively) and as next treatment in pH 3 (c, f, i respectively) where red numbers are the area of samples (the grid in the background is not calibrated, it is only for creating contrast between the samples and background).**


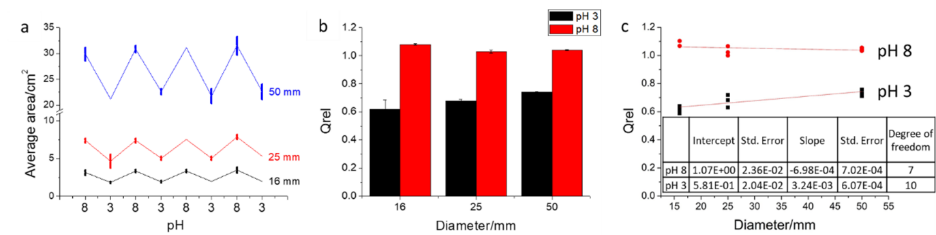


**S3 Fig. Average area of PASP-DAB disks of different initial sizes in pH 8 and pH 3 buffer solutions in 4 cycles (vertical line indicate standard error, and the lines between the points are just guides for the eye) (a), relative degree of swelling of PASP-DAB disks in pH 3 and pH 8 (b), relative degrees of swelling plotted as a function of initial diameter of dry cylinders where the table contains the data of the fitted linears (c).**

**Chemical analysis by FTIR**

The chemical analysis by NMR and FTIR of PSI, PASP and their modified versions is well documented in the literature.[8–12] Therefore, for the chemical analysis, only FTIR was used. The indirect evidence for the presence of crosslinks in PSI fibrous membranes was the dissolution test in DMF, where both the PSICYS and the PSIDAB crosslinks inside the fibers prevented them from dissolution. In this regard, the investigation by FT-IR was performed to further verify the crosslinking and the modification of PSI (S4A Fig). The characteristic absorption bands of imide rings in PSI can be seen at 1710 cm^‑1^ (asymmetric stretching vibration is attributed to the ν_CO_ of –(OC)_2_N–), 1391 cm^‑1^ (C–O bending vibration, δ) and 1355 cm^-1^ (stretching vibration, ν_C–N_ of –(OC)_2_N–) [13]. In the case of both PSICYS (S4B Fig) and PSIDAB (S4D Fig) the appearance of a shoulder on the peak at 1710 cm^‑1^ marks the vibration of the >N-C=O bond at 1636 cm^-1^ which is caused by modified succinimide units produced by crosslinking. Furthermore, there is another peak emerging at 1515cm^‑1^ also related to this modification [14]. Hydrolysis caused the same change in both types of samples. Compared to their precursor polymers, in the spectra of both PASPCYS (S4C Fig) and PASPDAB (S4E Fig) a new wide peak appeared at around 3320 cm^-1^ indicating new –OH groups formed from the hydrolyzed carboxyl groups on the polymer backbone (Fig 1b) [15]. Lastly, the reduction of the peak at 1710 cm^-1^ and the rise of the peak at 1662 cm^-1^ confirm the opening of succinimide rings.


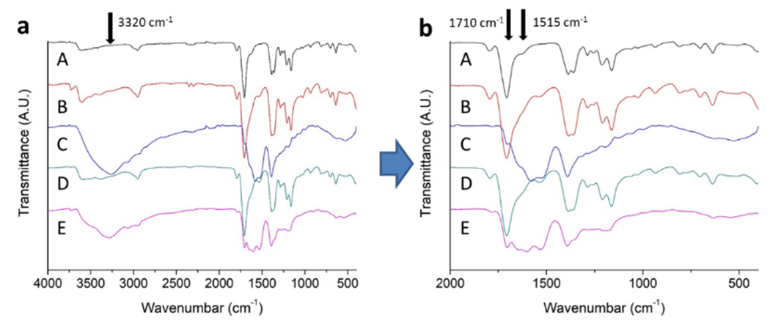


**S4 Fig. ATR-FTIR spectra of the synthesized samples (a) and an enlarged part of the said spectra (b), where the samples are labeled with capital letters as follows: PSI (A), PSICYS (B), PASPCYS (C), PSIDAB (D), PASPDAB (E).**


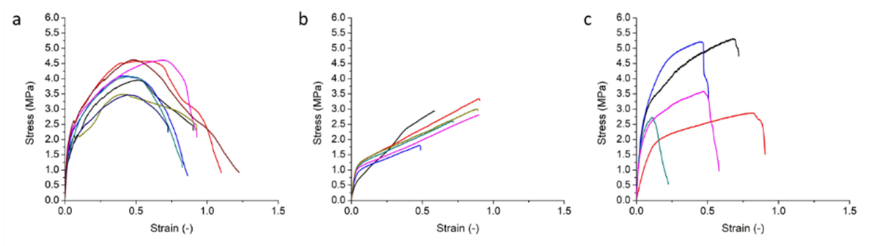


**S5 Fig. Stress strain curves of PSI (a), PSIDAB (b) and PSICYS (c) samples.**


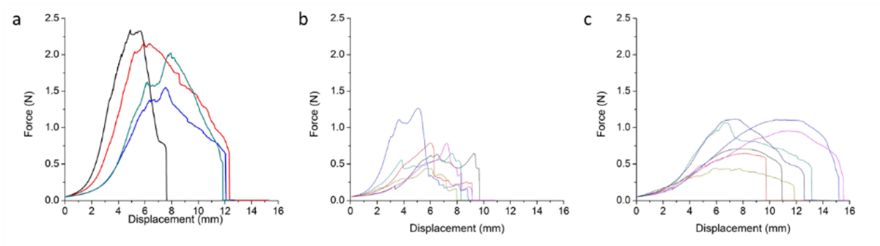


**S6 Fig. Force displacement curves measured as sutures were torn out (SIS model) from PSI (a), PSIDAB (b) and PSICYS (c) samples.**


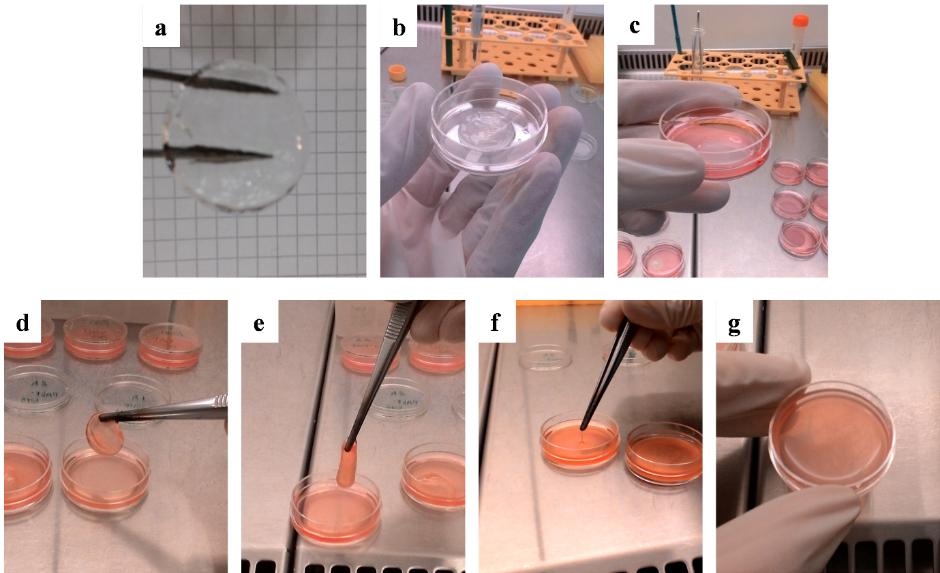


**S7 Fig. Dissolution of electrospun PASPCYS fibers in minimal essential media. The PASPCYS fibrous membranes stored in PBS (a, b). The degree of dissolution after 0 h (c), 24 h (d), 48 h (e), 72h (f) 96 h (g).**


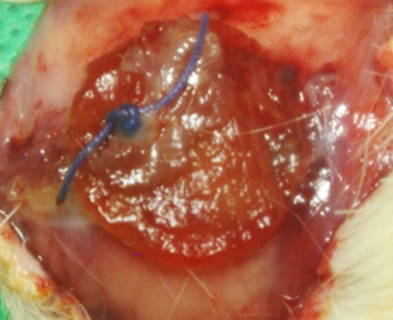


**S8 Fig. PSICYS sample 3 days after implantation.**

**
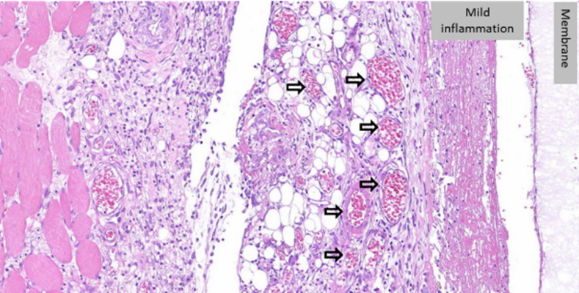
**

**S9 Fig. Histological slide of PSICYS sample in 3 day implantation, where empty black arrows indicate some of the blood vessels as part of the newly formed tissue.**


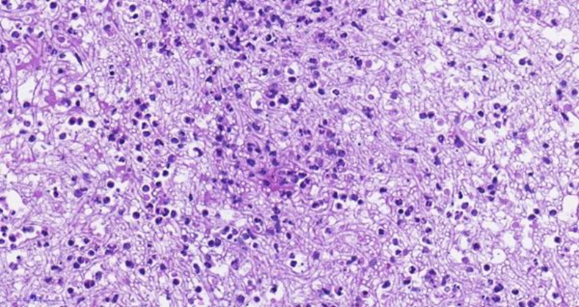


**S10 Fig. Close look on the inside of PSIDAB sample on a histological slide in 3 day implantation. Inflamatory cells including neutrophil granulocytes and lymphocytes infiltrate the membrane.**


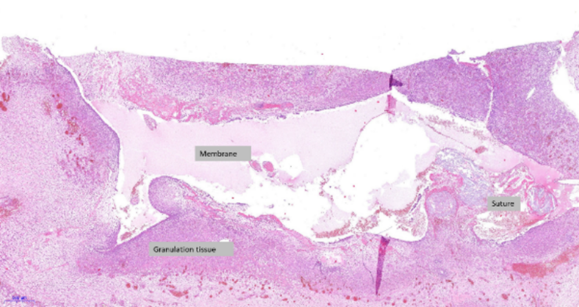


**S11 Fig. Histological slide of PSICYS sample in 7 day implantation.**


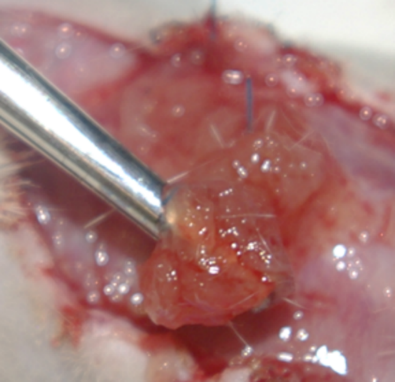


**S12 Fig. PSIDAB membrane after 7 days can be easily truncated with a tweezer.**


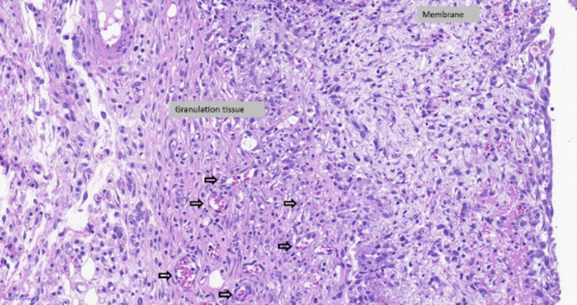


**S13 Fig. Histological slide of PSIDAB sample in 7 day implantation, where empty black arrows indicate some of the blood vessels as part of the newly formed granulation tissue.**

**References**

1. Piątkowski M, Pragłowska JR-, Raclavský K. Application of Poly ( aspartic acid ) and its Derivatives in Medicine and Pharmacy. Asian J Appl Sci. 2015;03: 718–723.

2. Krisch E, Messager L, Gyarmati B, Ravaine V, Szilágyi A. Redox- and pH-Responsive Nanogels Based on Thiolated Poly(aspartic acid). Macromol Mater Eng. 2016;301: 260–266. doi:10.1002/mame.201500119

3. Molnar K, Jedlovszky-Hajdu A, Zrinyi M, Jiang S, Agarwal S. Poly(amino acid)-Based Gel Fibers with pH Responsivity by Coaxial Reactive Electrospinning. Macromol Rapid Commun. 2017;201700147: 1700147–1700151. doi:10.1002/marc.201700147

4. Gyenes T, Torma V, Gyarmati B, Zrínyi M. Synthesis and swelling properties of novel pH-sensitive poly(aspartic acid) gels. Acta Biomater. 2008;4: 733–44. doi:10.1016/j.actbio.2007.12.004

5. Tanaka T, Hocker LO, Benedek GB. Spectrum of light scattered from a viscoelastic gel *. J Chem Phys. 1973;59: 5151–5159. doi:10.1063/1.1680734

6. Varga Z, Molnár K, Torma V, Zrínyi M. Kinetics of volume change of poly(succinimide) gels during hydrolysis and swelling. Phys Chem Chem Phys. 2010;12: 12670–12675. doi:10.1039/c0cp00527d

7. Li Y, Tanaka T, Yong L, Tanaka T. Kinetics of swelling and shrinking of gels. J Chem Phys. 1990;92: 1365–1371. doi:10.1063/1.458148

8. Krisch E, Gyarmati B, Barczikai D, Lapeyre V, Szilágyi BÁ, Ravaine V, et al. Poly(aspartic acid) hydrogels showing reversible volume change upon redox stimulus. Eur Polym J. 2018;105: 459–468. doi:10.1016/j.eurpolymj.2018.06.011

9. Szilágyi BÁ, Gyarmati B, Horvát G, Laki Á, Budai-Szűcs M, Csányi E, et al. The effect of thiol content on the gelation and mucoadhesion of thiolated poly(aspartic acid). Polym Int. 2017;66: 1538–1545. doi:10.1002/pi.5411

10. Juriga D, Laszlo I, Ludanyi K, Klebovich I, Chae CH, Zrinyi M. Kinetics of dopamine release from poly(aspartamide)-based prodrugs. Acta Biomater. 2018;76: 225–238. doi:10.1016/j.actbio.2018.06.030

11. Jalalvandi E, Shavandi A. Polysuccinimide and its derivatives: Degradable and water soluble polymers (review). Eur Polym J. 2018;109: 43–54. doi:10.1016/j.eurpolymj.2018.08.056

12. Molnar K, Jozsa B, Barczikai D, Krisch E, Puskas JE, Jedlovszky-Hajdu A. Plasma treatment as an effective tool for crosslinking of electrospun fibers. J Mol Liq. 2020;303: 112628–112636. doi:10.1016/j.molliq.2020.112628

13. Kim M, Shin SW, Lim CW, Kim J, Um SH, Kim D. Polyaspartamide-based graft copolymers encapsulating iron oxide nanoparticles for imaging and fluorescence labelling of immune cells. Biomater Sci. 2017. doi:10.1039/C6BM00763E

14. Shinoda H, Asou Y, Suetsugu A, Tanaka K. Synthesis and Characterization of Amphiphilic Biodegradable Copolymer, Poly(aspartic acid-co-lactic acid). Macromol Biosci. 2003;3: 34–43. doi:10.1002/mabi.200390007

15. Wang B, Jeon YS, Park HS, Kim YJ, Kim JH. Mussel-mimetic self-healing polyaspartamide derivative gel via boron-catechol interactions. Express Polym Lett. 2015;9: 799–808. doi:10.3144/expresspolymlett.2015.75
