# Supplementary figures and images for: Poly(amino acid) based fibrous membranes with tuneable in vivo biodegradation
Source: PLoS One. 2021 Aug 13;16(8):e0254843. doi: 10.1371/journal.pone.0254843 (PMC8362958; doi:10.1371/journal.pone.0254843)

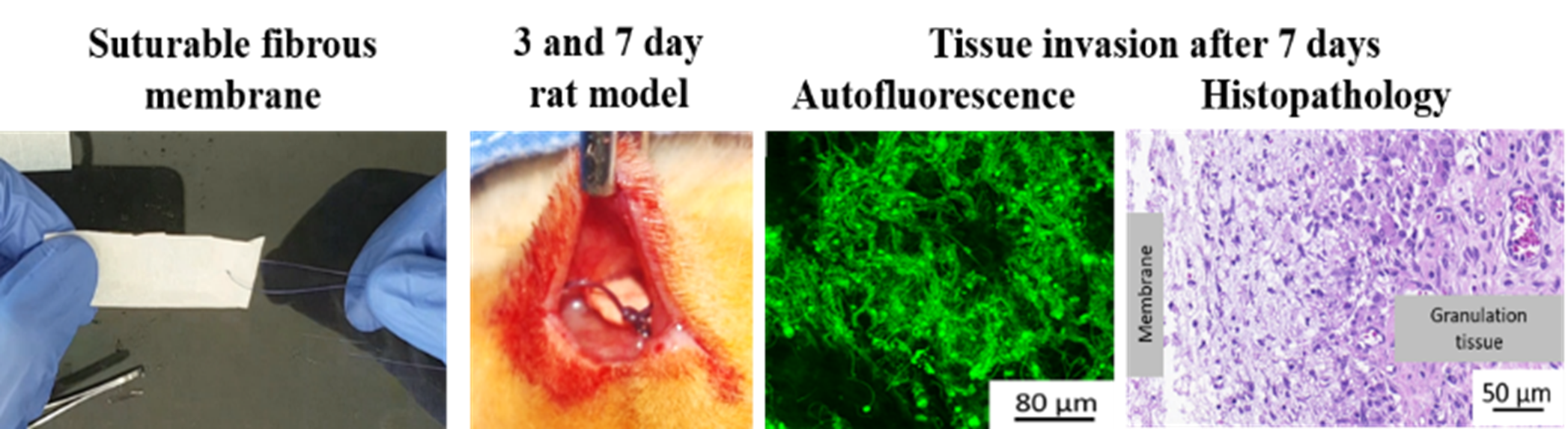

Supplement: S1 Graphical abstract — (TIF) [file pone.0254843.s002.tif]
